# A data-driven elucidation of the challenges in designing stable quinone derivatives for aqueous organic redox flow batteries: correlations between thermodynamics of chemical degradation and energy capacity metrics

**DOI:** 10.1080/14686996.2026.2698227

**Published:** 2026-07-16

**Authors:** Ning Lu, Shiyao Ju, Daniel Willimetz, Shengming Tang, Abhishek Khetan

**Affiliations:** a MODES, The Integrated Fuel & Chemical Science Centre, RWTH Aachen University, Aachen, Germany; b Fraunhofer Institute for Algorithms and Scientific Computing SCAI, Fraunhofer-Gesellschaft, Schloss Birlinghoven 1, Sankt Augustin, Germany

**Keywords:** Aqueous organic redox flow batteries, quinone stability, degradation, molecular design, high-throughput screening, durability, grid-scale energy storage

## Abstract

Aqueous organic redox flow batteries (AORFBs) offer significant potential for grid-scale storage of renewable energy. However, their commercial viability is often limited by the chemical instability of the organic electrolytes. Quantifying and assessing the effects of the various degradation mechanisms from a molecular design perspective is very challenging both experimentally and computationally. Here, we propose the use of simple thermodynamic descriptors for seven degradation mechanisms for a diverse virtual library of ca. 2000 monofunctionalized quinones built from seven core structures. In the process, we developed a cheminformatics-based workflow that can reliably and automatically generate reactants and mechanism-specific degradation products. Using DFT-calculated reaction Gibbs free energies as thermodynamic descriptors and a calibrated model for redox-potential prediction, we systematically analyse seven degradation mechanisms across this library. We find clear relationships between redox potential and degradation thermodynamics for six of the seven mechanisms, with opposite trend directions for oxidized and reduced forms, revealing stability – potential trade-offs that constrain molecular design. The analysis further shows how functional groups modulate degradation thermodynamics and thereby helps rationalize the relative stability of anthraquinone-based outliers. Finally, redox active molecules from recent experimental studies are evaluated within the proposed thermodynamics-based framework, and we comment on the implications of electrochemical reversibility of some critical degradation mechanisms. Overall, this work provides a physically motivated framework for the multi-objective screening of quinone-based AORFB electrolytes and helps clarify the design criteria needed to identify favourable molecular outliers.

## IMPACT STATEMENT

This work comprehensively analyses major quinone degradation mechanisms, reveals pathway-specific stability trade-offs, and identifies structural distortion and reversibility as critical yet previously overlooked stability descriptors.

The work presents the most comprehensive analysis to date of all major quinone degradation mechanisms, revealing pathway-specific trade-offs and identifying structural distortion and reversibility as critical, previously overlooked stability descriptors.

## GRAPHICAL ABSTRACT

**Figure:**
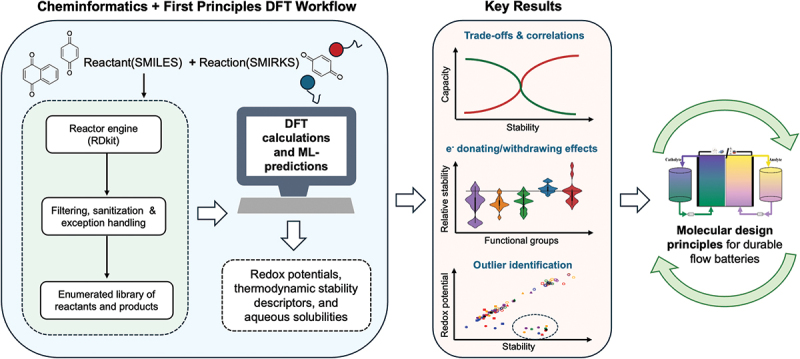

## Introduction

Redox flow batteries (RFBs) offer a promising and scalable solution for storing large amounts of electrical energy [1–3]. Their distinctive design allows the independent scaling of power and energy by adjusting the electrode active surface area and the concentration of redox-active materials (RAMs) in the electrolyte [4–6]. While vanadium redox flow batteries lead the market in terms of share [7], their limited natural availability and high cost of vanadium restrict their scalability and long-term viability [4,8,9]. As a result, the next-generation RFBs need to be developed that can operate durably for decades, offer high round-trip efficiency, respond rapidly to grid fluctuations, and are scalable for sustainable large-scale energy storage.

Aqueous organic redox flow batteries (AORFBs) present a greener and more cost-effective alternative to metal-ion-based redox flow batteries for intermittent energy storage [4,10–12]. The RAMs in AORFBs are organic molecules dissolved in aqueous electrolytes that ideally undergo reversible redox reactions at the electrodes during charge and discharge. For practical deployment in AORFBs, RAMs must simultaneously exhibit suitable redox potentials, high chemical and electrochemical stability [13,14], sufficient aqueous solubility [15–17], and low cost through feasible and scalable synthesis [18]. Among various candidates, quinones are some of the most extensively studied organic RAMs for AORFBs due to their tuneable redox potentials, favourable aqueous solubility, and fast redox kinetics [19–22]. The aromatic π-systems embedded in quinone structures contribute to their relatively high intrinsic stability and reversible electrochemical behaviour by providing delocalization of the radicals formed during redox processes [19].

From the point of view of commercial application, however, quinone-based molecules still exhibit insufficient stability [23,24]. They are prone to decomposition under aqueous conditions via various mechanisms, such as Michael addition [25–27], gem-diol formation [26,28,29], anthrone formation [30–32], tautomerization [33–35], disproportionation [36,37], or dimerization [29,33], thereby compromising their long-term electrochemical performance. The degradation of redox-active molecules can result in irreversible capacity loss, posing a significant challenge to their commercial application [25]. As a result, enhancing the stability of AORFB electrolytes has become an increasingly important focus in the field [7,19,20,25,38–40].

Over the past few years, extensive experimental investigations have deepened our understanding of the stability of quinone-based electrolytes in AORFBs [30,39]. Many efforts have focused on inhibiting degradation reactions [40–43], and achieving stable cycling under operational conditions [44]. Substitution strategies such as phosphonate-functionalization [8], sulfonation [45], and amino-acid zwitterionic modification [39] have been employed to suppress side reactions. Structural strategies such as π-conjugation extension [19], asymmetric substitution, and branching [41] have proven effective in increasing redox reversibility and molecular robustness. Notably, full substitution of reactive positions on hydroquinone rings has been shown to significantly mitigate Michael addition reactions, leading to decay rates as low as 0.45%/day in symmetric cell tests [46]. In alkaline media, particularly with anthraquinone derivatives, stable cycling over more than 1000 cycles has been achieved, with capacity fade rates below 0.01% per cycle [30]. Collectively, these experimental advancements inspire molecular design of stable quinone-hydroquinone pairs, thereby reducing the gap between laboratory demonstrations and practical, scalable AORFB applications [28].

Recent computational studies have provided detailed insights into the stability of quinone-based molecules in AORFBs [47,48]. These investigations have elucidated how specific functional groups and molecular structures influence several degradation reactions. For example, tertiary ammonium groups in biphenol derivatives can significantly improve stability by inhibiting Michael addition reactions [48], while sulfonic acid groups can promote degradation in acidic media, and methyl groups can enhance chemical inertness [47]. The position of functional groups on the quinone core structure (ortho vs. para) further modulates stability, highlighting the critical role of molecular architecture in determining susceptibility to tautomerization [34], Michael addition [25], and gem-diol formation [28]. Collectively, these studies demonstrate that functionalization strategies and core structure design can be leveraged to improve the chemical stability of quinone-based redox-active molecules.

While several large-scale computational efforts have explored subsets of quinone derivatives [28,33,49], most studies have focused on individual degradation mechanisms or specific molecular core structures. For example, Tabor et al. created a large database of quinone-based structures and analysed their stability toward Michael addition and gem-diol formation, finding that very few molecules simultaneously achieve a high reduction potential and thermodynamic protection against these degradation pathways [28]. Similarly, Tong et al. demonstrated that dimerization and tailored functional groups can enhance stability by mitigating tautomerization [33]. These studies highlight the importance of both core-structure selection and functionalization in improving quinone robustness; however, a comprehensive virtual library spanning diverse quinone structures and multiple degradation pathways is still lacking.

In this work, we construct a comprehensive virtual library of 1,956 quinone-derived molecules by systematically generating reactants and their corresponding degradation products using a combined RDKit-[50] and SMIRKS-[51] based approach, enabling consistent coverage of different core structures, functionalization, and degradation pathways reported in the literature. Building on this library, we provide a thermodynamic description of quinone degradation by calculating the Gibbs free energy of reaction for each mechanism, which serves as a simple and physically motivated descriptor of a molecule’s susceptibility to degradation. We then investigate the relationship between redox potential and stability across several degradation pathways to identify structure-property trends, which have not yet been reported and that must be considered in the rational design of molecules. We also discuss the absence of a relationship between stability and solubility. Finally, we rationalize our findings in the context of experimentally extracted metrics of stability, including an analysis of outliers, and propose new degrees of freedom for exploring the design space.

## Methods

### Generation of initial structures

**Figure 1.**
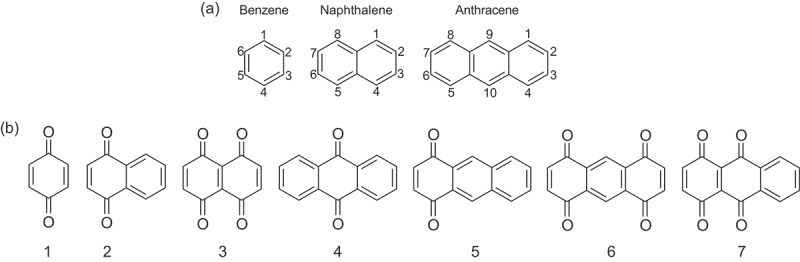
(a) Carbon atom numbering scheme used to define substitution positions on benzene, naphthalene, and anthracene. (b) Quinone core structures investigated in this study (1–7).

To investigate the electrochemical properties of candidate quinones, we constructed a virtual molecular library starting from seven quinone core structures derived from aromatic backbones (benzene, naphthalene, and anthracene) (Figure 1(a)). The core structures are labelled 1–7 (Figure 1(b)). They are generated by installing carbonyl (C=O) groups at different combinations of substitution sites on the corresponding backbones (Figure 1(a)). For example, core structure 1 is formed by placing carbonyl groups at sites 1 and 4 of the benzene backbone, and the remaining core structures are defined analogously. Based on the number of carbonyl pairs, the core structures can be divided into two classes: single-pair (SO) core structures (1, 2, 4, and 5), which contain one carbonyl pair, and double-pair (DO) core structures (3, 6, and 7), which contain two carbonyl pairs.

**Figure 2.**
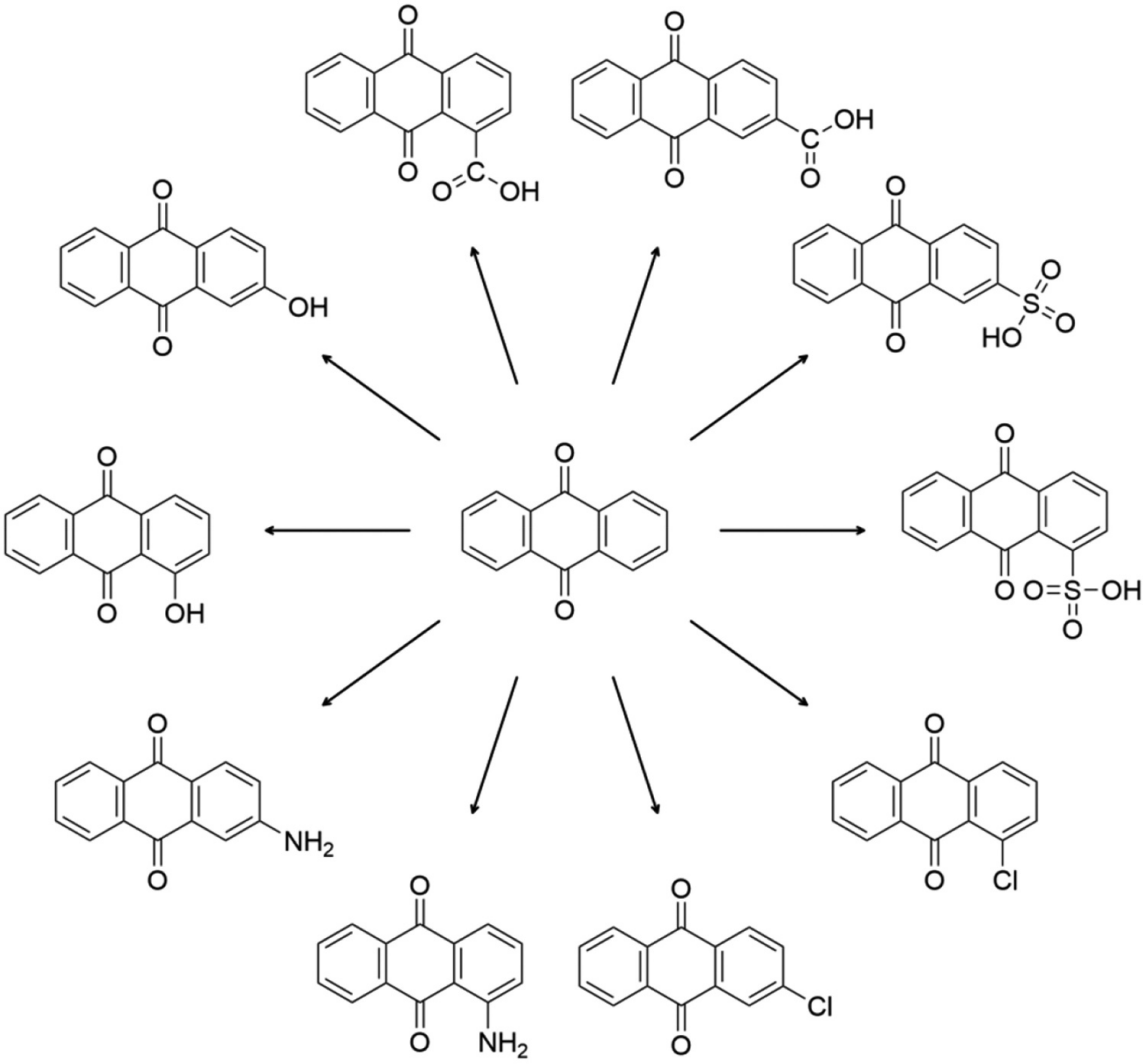
A schematic representation of the anthraquinone functionalization applied during the enumeration process for generating the virtual molecular library.

It is well established that both the type [52] and position [53] of functional groups can substantially affect the redox thermodynamics and kinetics of quinones, including functional group-dependent shifts in redox potential [15,54], and changes in proton-coupled electron transfer (PCET) behaviour in aqueous media. To build a chemically tractable yet electrochemically relevant screening library for aqueous quinone-based redox electrolytes, we systematically functionalized each core structure with five representative functional groups (-SO_3_H, -COOH, -NH_2_, -OH, and -Cl) to span a broad range of electronic effects (electron-withdrawing vs. electron-donating) and battery-relevant physicochemical properties, notably ionizability, hydrogen-bonding capability, and enhanced aqueous solubility. In this set, -SO_3_H and -COOH serve as strongly solubilizing/ionizable electron-withdrawing groups that are commonly used to mitigate precipitation and enable high active-material concentrations, whereas -NH_2_ and -OH provide electron-donating and hydrogen-bonding functionality that can tune redox potential and intermolecular interactions in aqueous electrolytes. Chlorine was selected as a small, non-ionizable electron-withdrawing functional group and as a practical representative of halogen substitution (synthetically accessible and electronically well-defined). An illustrative example of the functionalization workflow for anthraquinone is shown in Figure 2. Functionalized derivatives were exhaustively enumerated using RDKit [50,55,56] (via Python [57]). In total, the seven quinone core structures yield 87 functionalized reactant quinones. In our virtual library, each entry is defined by its structural representation, i.e. a SMILES string.

We then compiled several degradation mechanisms from the literature (Table 1) to enumerate degradation products alongside the main redox reaction [49]. In acidic aqueous solutions, quinones typically undergo two-electron, proton-coupled redox reactions in redox flow batteries [58]. The main electrochemical reaction is listed in the first row of Table 1. The degradation mechanisms can differ depending on whether the quinone is in its oxidized or reduced state. For the oxidized (quinone) form, reported pathways include Michael addition [26,30,59], gem-diol formation [26,28], proto-desulfonation [60], and anthrone formation [30,40]. Pathways involving the reduced (quinol) form include tautomerization [46], disproportionation [40,61], and dimerization [35,36,62].

**Table 1.** The schemes of the reaction mechanism.

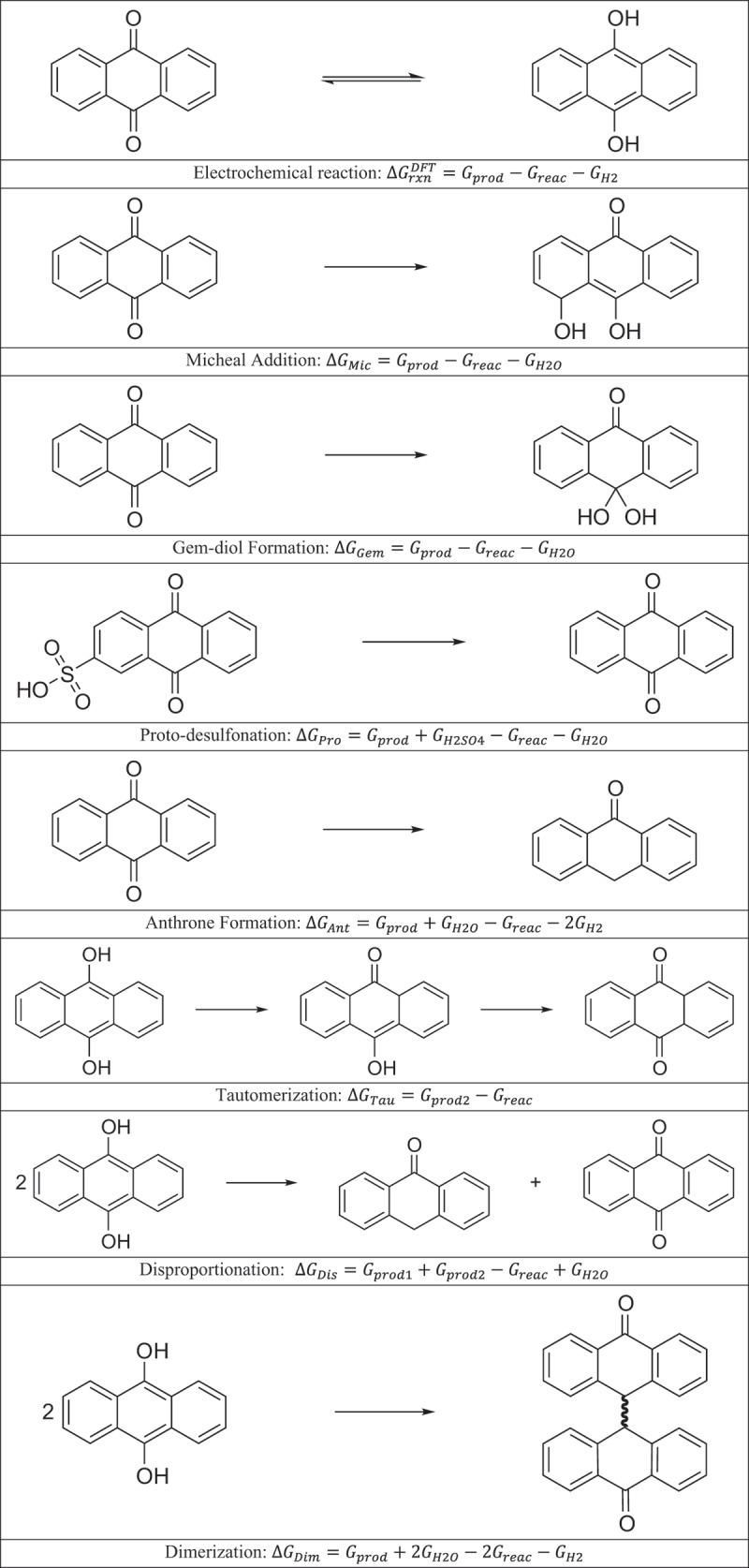

### Extension of the virtual library using reaction-based enumeration

The next step is to extend the virtual library to enumerate the SMILES strings of all products from the degradation reactions shown in Table 1, and this step comprises two parts: (i) designing SMIRKS^62^ reaction templates to encode the transformation rules, and (ii) implementing a SMIRKS-based enumeration algorithm to generate product SMILES along with exception handling for incorrect transformations.

To represent each reaction type with a manageable number of SMIRKS templates, we restricted the design to the minimal transforming substructures-i.e. only the atoms and bonds that change during the reaction-while leaving nonreacting parts unspecified. For the main electrochemical reaction, five distinct SMIRKS templates were created, as shown in the first column of Table 2. The second column of Table 2 illustrates how these five SMIRKS templates match quinone compounds with different core structures. The key difference among these templates is whether the reacting carbon atoms are aromatic. Note that in the corresponding RDKit depictions, aromaticity is explicitly reflected in the drawing style: aromatic atoms/bonds are rendered with aromatic bond representations (e.g. dashed/alternating bonds), whereas non-aromatic (Kekulé) structures are shown with explicit single and double bonds. When an input reactant matches a given SMIRKS template, all possible products consistent with that template are enumerated. This SMIRKS design strategy, which distinguishes aromatic from non-aromatic carbons, effectively captures quinone redox reactions and applies to other reaction types as well. In total, we designed 35 SMIRKS templates to represent the eight reaction types considered in this work, comprising the seven degradation reactions and the main electrochemical redox reaction listed in Table 1. The additional SMIRKS templates corresponding to the degradation reactions are provided in the Supplementary Information (Table S1).

**Table 2.** Example of five SMIRKS templates corresponding to the main electrochemical reaction.

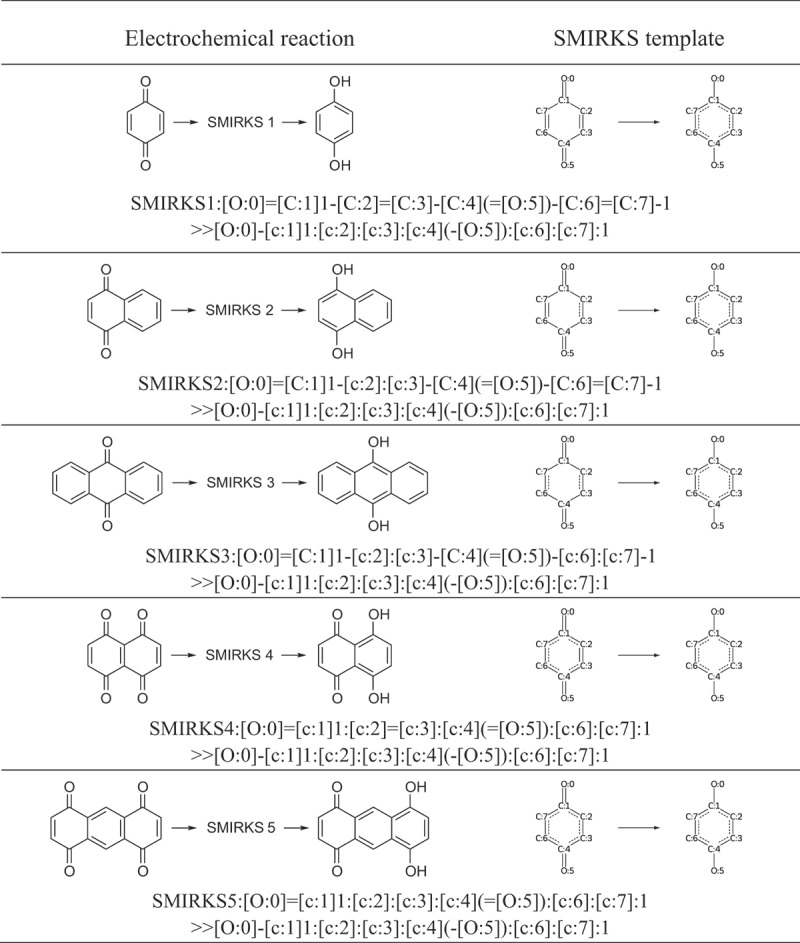

Next, the reactant SMILES are processed by an RDKit-based reactor module, which applies the relevant SMIRKS templates to enumerate all possible products. The resulting structures are then filtered by removing duplicates and discarding invalid molecules that fail RDKit sanitization (e.g. valence, aromaticity, and charge/valence-state consistency checks). Finally, the output SMILES are organized by reaction type and exported to separate CSV files.

In summary, using this procedure, we first systematically enumerated 113 functionalized quinols from the 87 quinones. After further enumeration using degradation reactions associated with either quinones or quinols, the virtual library comprises 1,956 unique structures. This set provides a reliable and comprehensive foundation for subsequent computational analyses and mechanistic investigations.

### Computational methods

All electronic structure calculations were performed using the ORCA (v5.0.3) software [63]. Geometry optimizations were carried out using the B3LYP exchange-correlation functional [64] and the 6–311++G(d,p) basis set, with implicit solvation treated using the conductor-like polarizable continuum model (CPCM) for water [65–67]. Optimization convergence was achieved when the maximum force was below3 × 10^−4^ Hartree/Bohr, and the root mean square force was below 1 × 10^−4^ Hartree/Bohr. Gibbs free energies were also obtained under CPCM by including entropic corrections and zero-point energy contributions. We estimated the aqueous solubility of the reactant molecules using AqSolPred v1.0 [68], a state-of-the-art consensus ML model trained on AqSolDB, a large open-access curated dataset of experimentally measured aqueous solubilities [69].

For correlating degradation thermodynamics with aqueous solubility, we used either the reduced- or oxidized-state of the molecule, depending on which state the degradation was evaluated. Aqueous solubility was estimated using AqSolPred v1.0 [68], a consensus ML model trained on AqSolDB, which is a curated open-access dataset of experimentally measured aqueous solubilities [69]. This ML-predicted solubility is reported in logS units, i.e. the base-10 logarithm of the molar aqueous solubility, at 25°C. AqSolPred is one of the state-of-the-art models for predicting aqueous solubilities of drug-like molecules with a logS error < 0.4 units. It simply uses the SMILES representations of the molecules for a direct prediction of solubility.

In addition to logS, the CPCM-based solvation free energy [66], $\Delta G_{\mathrm{sol}}$, was used as an approximate descriptor representing the aqueous solvation contribution to the solubility. $\Delta G_{\mathrm{sol}}$ was calculated as the difference between the Gibbs free energy obtained with CPCM water solvation and the corresponding gas-phase Gibbs free energy:

(1) $$\Delta G_{\mathrm{sol}}=G_{\mathrm{CPCM}}-G_{\mathrm{gas}}$$

In their current usage, neither logS nor $\Delta G_{\mathrm{sol}}$ explicitly accounts for pH-dependent speciation, supporting-electrolyte composition, salt formation, concentration-dependent aggregation, or other condensed-phase effects that may influence experimentally measured solubility in AORFB electrolytes. Therefore, both quantities should be interpreted as descriptors for high-level screening rather than a direct prediction of experimentally validated solubilities under specific electrolyte formulations.

The redox potential is defined as the reaction Gibbs free energy change per unit charge transferred [49,70,71]. The calculated reaction energies are often calibrated empirically against experimental redox potentials using a simplified linear relationship (Eq. 2).

(2) $$E_{\left(SHE\right)\;}^{0}=\;m\left(G_{i}^{DFT}\left(Q\right)\;-\;G_{i}^{DFT}\left(QH_{2}\right)-G_{i}^{DFT}\left(H_{2}\right)\right)+c$$

In this equation, *m* and *c* denote the slope and intercept of the linear calibration model, respectively, and $G_{i}^{DFT}$ represents the Gibbs free energy [71]. Therefore, an empirical linear calibration was performed between the experimental redox potentials of 25 quinone couples (Supporting Information Table S2) and their calculated reaction Gibbs free energies [21,25,28,72]. The resulting calibration equation for predicting redox potentials versus SHE at pH = 0 is given in Eq. (3) and shows excellent agreement with experiment (R^2^ = 0.98; MAE = 33 mV; Supporting Information Figure S1).

(3) $$E^{0}=\;-0.48\left[\Delta G_{rxn}^{DFT}\right]+0.18$$

Here $E^{\circ }$ (V) represents the redox potential, and $\Delta G_{rxn}^{DFT}$(eV) is defined as the Gibbs free energy difference between the products and reactants of the electrochemical reaction.

In our study, we use $\;\Delta G_{\mathrm{stability}}$, defined as the Gibbs free energy difference between the products and reactants of different degradation mechanisms, as a thermodynamic descriptor of degradation susceptibility in aqueous solution. Accordingly, for a given degradation reaction, a more negative $\;\Delta G_{\mathrm{stability}}$ indicates a stronger thermodynamic driving force toward instability, implying a higher propensity for decomposition [73]. Although $\;\Delta G_{\mathrm{stability}}$ does not capture kinetic barriers or reaction rates, it offers a physically motivated and internally consistent metric for comparing the intrinsic thermodynamic tendency of different molecules to degrade.

## Results and discussion

**Figure 3.**
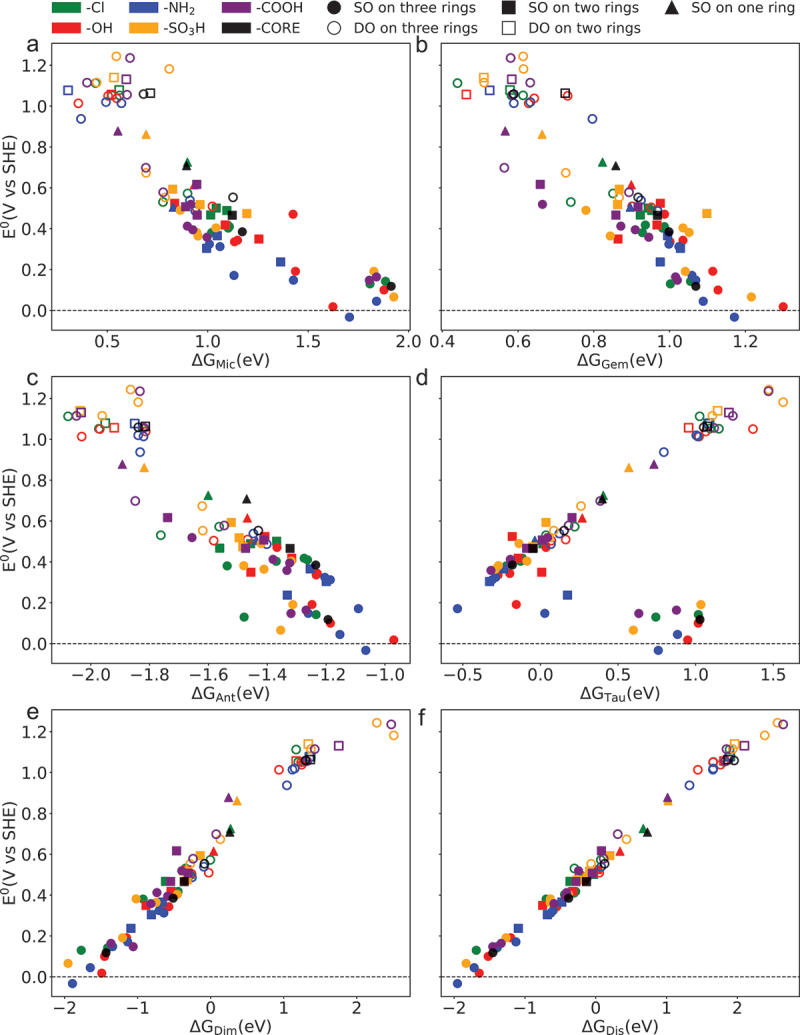
Correlation between predicted half-cell redox potential and $\Delta G_{\mathrm{reaction}\;\mathrm{type}}$ for six degradation mechanisms (excluding proto-desulfonation) of quinone-hydroquinone pairs.

We first investigated the relationship between redox potential and thermodynamic stability for the various degradation mechanisms, as shown in Figure 3. In these plots, $\Delta G_{\mathrm{reaction}\_\mathrm{type}}$ denotes the thermodynamic stability descriptor for a specific reaction, as defined in Table 1. The various molecules in the dataset are colour-coded according to the type of functional group and shape-coded according to the number of rings. Furthermore, filled symbols represent molecules with a single pair of C=O groups, whereas molecules with two pairs of C=O groups. Figure 3(a–c) ($\Delta G_{\mathrm{Mic}}$), ($\Delta G_{\mathrm{Gem}}$), ($\Delta G_{\mathrm{Ant}}$) show the correlations for the oxidized (quinone) forms, and Figure 3(d–f) ($\Delta G_{\mathrm{Tau}}$), ($\Delta G_{\mathrm{Dim}}$), ($\Delta G_{\mathrm{Dis}}$) show those for the reduced (quinol) forms.

First, it can be observed that, for every mechanism, there is a very clear correlation between $\Delta G_{\mathrm{reaction}\_\mathrm{type}}$ and $E^{\circ }$. This correlation indicates that, for planar molecules with single functional groups, such as those considered in our dataset, the electron-donating/withdrawing character has a decisive influence on thermodynamic stability, much like it does on redox potential. It is also clearly observable that molecules with two pairs of C=O groups have higher redox potentials than those with a single pair (Fig. S2) [28,74]. However, the quinone (oxidized) and quinol (reduced) forms exhibit opposite trends. For Michael addition, gem-diol formation, and anthrone formation, a more positive $\Delta G_{\mathrm{reaction}\_\mathrm{type}}$ (i.e. greater thermodynamic resistance to degradation) is generally associated with a lower redox potential, indicating a favourable design space for anolytes. This trend is consistent with Tabor *et al*.’s analysis of Michael addition degradation in quinones [28], which links higher $E^{\circ }$ to increased electrophilicity of the quinone core, thus resulting in greater thermodynamic susceptibility to nucleophilic attack.

In contrast, tautomerization, disproportionation, and dimerization show the reverse behaviour, with lower redox potentials corresponding to more negative $\Delta G_{\mathrm{reaction}\_\mathrm{type}}$, indicating a trade-off that must be balanced when designing anolytes. In the case of tautomerization, the positive correlation is consistent with the study by Tong et al. [33] on reduced naphthoquinones, in which compounds with lower reduction potentials were found to be more prone to tautomerization. Notably, we also find a small subset of systematic outliers that deviate from the dominant trend, motivating a closer structure-based analysis in the following section.

For protodesulfonation, no clear correlation between redox potential and $\Delta G_{\mathrm{reaction}\;\mathrm{type}}$ is observed (Supporting Information Figure S3). A likely reason is that this reaction is governed primarily by the local electronic environment of the sulfonic acid group rather than by the overall electrophilicity of the quinone core. Therefore, this mechanism is not discussed further.

Overall, these observations indicate that both redox potential and degradation thermodynamics are strongly dependent on electron density changes induced by functional groups. A cursory look at the quinol forms would suggest that designing stable, low-potential anolytes is a futile task. However, thermodynamic susceptibility to degradation may fortunately not translate directly into long-term instability. A striking example of this is the recently demonstrated electrochemical reversibility of the disproportionation/dimerization reactions in the case of 2,6-dihydroxy-anthraquinone (DHAQ) in alkaline media [36]. In this study, Jing et al. were able to demonstrate non-invasive regeneration of the desired redox-active components by stimulating oxidative processes using an externally controlled potential that was only slightly outside the operating limits. The authors further confirmed the same regeneration in the case of anthraquinone-2,7-disulfonic acid (AQDS) in acidic media. These promising evidences point to the need to develop new stability descriptors that capture the reversibility of such degradation reactions rather than one-directional thermodynamic susceptibility. Another such example is the case of tautomerization (Figure 3(d)), in which a small outlier subset appears in the lower-right region of the plot, deviating from the main correlation line. To investigate these deviations in more detail, we first examined the molecular structures corresponding to the outliers and found that they are exclusively associated with anthraquinone cores (Figure 1(b), structure 4) and their functionalized derivatives. Our thermodynamic analysis predicts this subset of anthraquinone anolytes to be relatively resistant to tautomerization, which is consistent with numerous literature studies that have collectively established anthraquinones as benchmark molecules [43,44,61].

To rationalize the enhanced thermodynamic resistance of the anthraquinone-derived outliers to tautomerization, we investigated another metric of stability, i.e. structural distortion of the molecules upon reaction. The structural distortion was computed using the carbon-skeleton RMSD between DFT-optimized reactant and product geometries with the LS-align (Rigid-LS-align) [75] tool. For this analysis, we evaluated the four single-carbonyl parent core structures (1, 2, 4, 5) shown in Figure 1(b). Single-carbonyl structures were selected in order to enable a fair comparison and avoid the effects of intramolecular hydrogen bonding. The RMSD values for tautomerization were 0.213, 26.316, 56.432, and 37.742 Å for structures 1, 2, 4, and 5, respectively. Clearly, structure 4 shows the largest geometric rearrangement, consistent with a higher reaction Gibbs free energy and a less favourable tautomerization process, which plausibly explains its deviation from the main trend. The RMSD values for the cores with double carbonyls, namely structures 3, 6, and 7, are 22.194, 6.989, and 49.843 Å. Despite having relatively high RMSD values, they do not appear as outliers because their redox potentials are also high. Taken together, these observations indicate that tautomerization thermodynamics is governed not only by electron-density effects but also by the structural distortion of the resulting tautomerized product. Accordingly, thermodynamic screening should be interpreted as providing a baseline metric that would benefit from complementary kinetic and geometrical considerations.

### Effect of the functional groups on stability against specific degradation reactions

To assess functional group effects on quinone stability, we defined, for each degradation mechanism, the relative free energy difference ($\Delta \Delta G_{\mathrm{reaction}\_\mathrm{type}}$) between the reaction Gibbs free energies of each functionalized derivative ($\Delta G_{\mathrm{reactio}n_{\mathrm{type}}}^{\mathrm{functionalized}}$) and that of its corresponding parent core structure ($\Delta G_{re\mathrm{action}\_\mathrm{type}}^{\mathrm{core}}$), as follows:

(4) $$\Delta \Delta G_{\mathrm{reaction}\_\mathrm{type}}=\Delta G_{re\mathrm{action}\_\mathrm{type}}^{fu\mathrm{nctionalized}}-\Delta G_{re\mathrm{action}\_\mathrm{type}}^{\mathrm{core}}$$

**Figure 4.**
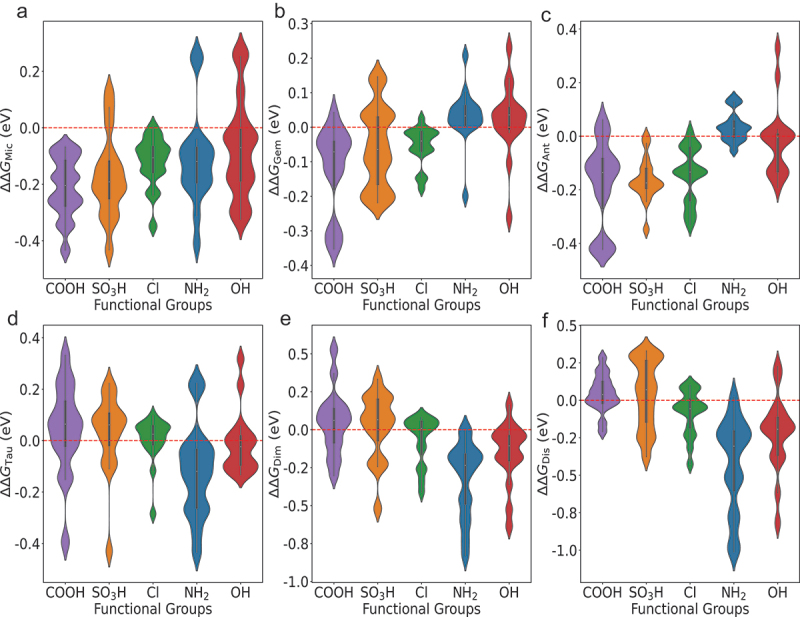
Effect of functional group on the stability of different degradation mechanisms.

A larger $\Delta \Delta G_{\mathrm{reaction}\_\mathrm{type}}$ indicates greater thermodynamic resistance of the functionalized derivative toward the corresponding degradation pathway relative to the parent core structure. We then analysed functional group effects across the various degradation reactions using this descriptor, as shown in the violin plots in Figure 4. The corresponding detailed, core-wise decomposition of $\Delta \Delta G_{\mathrm{reaction}\_\mathrm{type}}$ for each functional group and degradation mechanism, enabling direct comparison both within and across core structures, is provided in the Supporting Information (Figs. S4-S9).

It is not surprising that, for Michael addition, gem-diol formation, and anthrone formation, the trends are broadly similar, with the electron-donating (-OH, -NH_2_) functionalized cores generally showing higher values of $\Delta \Delta G_{\mathrm{reaction}\_\mathrm{type}}$ than the electron-withdrawing (-SO_3_H, -COOH) functionalized cores. Despite this, there are again striking differences with important consequences for molecular design. For example, for Michael addition (Figure 4(a)) and anthrone formation (Figure 4(c)), nearly all the considered functional groups lead to decreased stability, whereas for gem-diol formation (Figure 4(b)), a significant number of -SO_3_H, -OH, and -NH_2_-functionalized molecules exhibit higher stability than their parent cores. Nevertheless, there are fortunate exceptions and outliers, for example, naphthoquinone and anthraquinone cores seem to achieve higher stability across all degradation mechanisms when functionalized with -NH_2_ (Figs. S4–S6 in the Supporting Information). As expected, tautomerization, disproportionation, and dimerization show the opposite dependence on functional groups (Figure 4(d–f)). For all three mechanisms, the electron-withdrawing -COOH, -SO_3_H, and -Cl groups are broadly associated with positive $\Delta \Delta G_{\mathrm{reaction}\;\mathrm{type}}$ values, whereas -OH and -NH_2_ lie mainly below zero. A closer look at the more detailed distributions (Figs. S7–S9) again shows a large degree of coupling between the functional group and the core type, because even the same functional group has very different effects on benzo-, naphtho-, and anthraquinone cores.

While the overall landscape of quinone thermodynamic stability suffers from the bespoke trade-offs, a fortunate observation is that the range over which stability is influenced extends in both the positive and the negative direction. This implies that there may still be functional groups (or a combination of them, as discussed in section 3.3) that can be employed to balance these trade-offs.

These results have direct implications for molecular design and screening. Because functional group effects are pathway-specific and may reverse across mechanisms, no single functional group rule is universally favourable. Instead, functional group effects should be assessed by considering multiple degradation pathways alongside redox properties.

### Relations between stability and solubility descriptors

**Figure 5.**
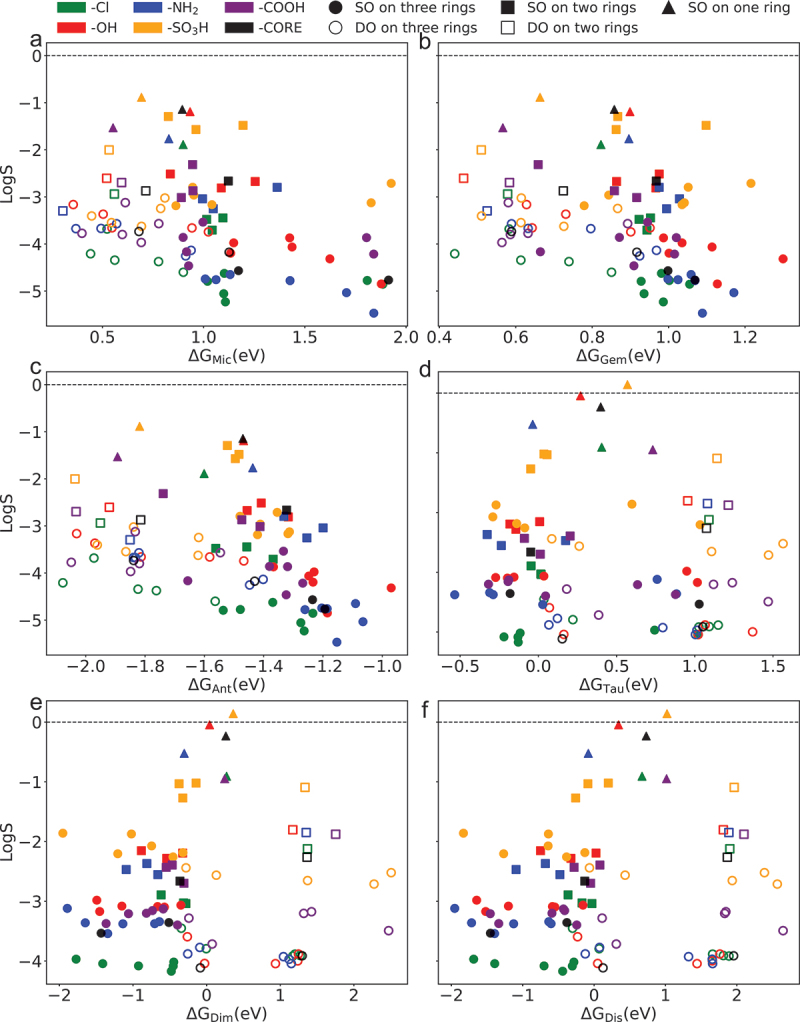
The relationship between the ML-predicted solubility (AqSolpred) and the reaction Gibbs free energy of the six degradation mechanisms.

Next, we examined the relationship between thermodynamic stability and two descriptors relevant to aqueous solubility: (a) the ML-predicted aqueous solubility (logS) and (b) the solvation free energy ($\Delta G_{\mathrm{sol}}$). Because aqueous solubility depends not only on solvation but also on intermolecular packing, aggregation, and other condensed-phase effects, $\Delta G_{\mathrm{sol}}$ is treated here as a complementary descriptor rather than a direct surrogate for logS. Figure 5 reveals weak and highly dispersed relationships between predicted aqueous solubility and $\Delta G_{\mathrm{reaction}\;\mathrm{type}}$ across all six degradation pathways. Although slight visual differences between the pathway-specific distributions may be present, no clear or robust monotonic trend can be identified for any individual mechanism. The corresponding analysis based on solvation free energy (Supporting Information, Figure S10) leads to the same general conclusion. The relationship between $\Delta G_{\mathrm{sol}}$ and $\Delta G_{\mathrm{reaction}\;\mathrm{type}}$ is likewise weak and broadly scattered, and no robust stability-dependent trend can be identified across the degradation pathways.

More broadly, the ML-predicted aqueous compatibility is governed more strongly by structural features, particularly ring count and carbonyl count. In comparison, functional group-specific effects are less clearly resolved. Overall, these results do not support a clear relationship between degradation thermodynamics and aqueous compatibility in the present dataset. It should be noted, however, that even state-of-the-art solubility prediction models do not have well-established uncertainty estimates [76,77], and their predictions can often be erroneous by several orders of magnitude. In any case, the absence of any specific correlation bodes well for the molecular design space.

### Approximate metrics of stability from experimentally studied molecules and their thermodynamic stability evaluation

A solid validation of our approach would ideally involve verification of the proposed stability descriptors against experimental stability data. As a starting point, we compiled a representative collection of quinone-based molecules reported in the literature as comparatively stable (Fig. S11) [19,20,26,30,39,40,44,46,78–80], thereby placing the present work in the context of experimentally studied compounds with promising electrochemical performance. Their reported performance was extracted in terms of various durability metrics, such as capacity retention, cycle number, energy efficiency, etc., as tabulated in Table S3 in the Supporting Information. A cursory look at the sparseness of Table S3 shows that the reported systems were tested under different solvent chemistries, pH, active-molecule concentrations, and current densities. This, combined with the fact that their stability can only be interpreted qualitatively (as opposed to being inferred quantitatively) and with the availability of disparate durability metrics, makes a direct comparison between theory and experiment very challenging. To the best of our knowledge, there are no experimental data on the individual reactions considered in our work that would enable a direct comparison between experimentally observed reaction rates and computed thermodynamic descriptors.

Nevertheless, most of the literature molecules collected align well with the broad stability-potential correlations identified in our analysis, with only a few notable outliers, as seen in Figure S12 in the Supporting Information. A large fraction of the experimentally collected molecules has two or more functional groups, which entail intramolecular interactions and effects beyond simple electron donation/withdrawal. On closer inspection, we find that the more outlying molecules are multi-functionalized species, which points to multi-functionalization as another degree of freedom that warrants further exploration. The same has also been evidenced in recent literature, for example, in the study by Alfaraidi et al. [80], who showed that combining a neighbouring -NH_2_ group with an ether-containing functional group (-O-CH(CH_3_)-COOH) in an anthraquinone electrolyte can simultaneously suppress side-chain loss and anthrone formation while maintaining high solubility. They reported an extremely low-capacity fade rate of 0.01%/day and a very high Coulombic efficiency of >99.8%.

## Conclusion

In this work, we performed a systematic thermodynamic analysis of multiple degradation pathways in quinone – quinol redox couples and established clear structure – property relationships linking redox potential, functionalization, and stability. Across all investigated mechanisms, strong correlations were identified between the reaction Gibbs free energy of various degradation reactions and the redox potential, confirming that electron-donating and electron-withdrawing effects govern both electrochemical properties and thermodynamic susceptibility to degradation. Notably, oxidized (quinone) and reduced (quinol) forms exhibit fundamentally different trends, highlighting an intrinsic asymmetry in the stability landscape that must be considered in molecular design.

For Michael addition, gem-diol formation, and anthrone formation, higher redox potentials correlate with increased thermodynamic susceptibility, consistent with enhanced electrophilicity of the quinone core. In contrast, tautomerization, disproportionation, and dimerization display the opposite trend, leading to inherent trade-offs between achieving low redox potential and maintaining thermodynamic stability. These competing relationships demonstrate that optimizing quinone-based anolytes cannot rely on a single descriptor but instead requires a multi-objective perspective that accounts for multiple degradation pathways simultaneously.

A key finding of this work is the identification of systematic outliers, particularly among anthraquinone derivatives, which exhibit enhanced resistance to tautomerization despite their redox characteristics. Detailed structural analysis revealed that this behaviour is linked not only to electronic effects but also to significant geometric distortion upon reaction, as quantified by RMSD metrics. These results emphasize that molecular stability is governed by a complex interplay of electronic, structural, and functionalization effects and that rational design must navigate competing trends across multiple reaction pathways.

Functional group effects were shown to be strongly dependent on the reaction-type and highly coupled to the underlying quinone core. While electron-donating groups generally stabilize against nucleophilic degradation pathways, they tend to destabilize reduced-form reactions, with the opposite behaviour observed for electron-withdrawing substituents. Importantly, no universally beneficial functional group was identified, reinforcing the need for balanced, context-specific design strategies. Nevertheless, the broad distribution of functional group effects in both stabilizing and destabilizing directions suggests that careful functionalization – and particularly multi-functionalization – offers viable routes to mitigate these trade-offs.

In contrast, the relationship between thermodynamic stability and aqueous compatibility (as described by ML-predicted solubility and solvation free energy) was found to be weak and non-systematic. This lack of correlation indicates that stability and solubility can, to a first approximation, be tuned independently, thereby expanding the accessible design space for aqueous organic redox flow battery electrolytes.

Comparison with experimentally studied quinone systems further supports the qualitative validity of the proposed descriptors, despite the challenges associated with heterogeneous experimental conditions and the absence of direct kinetic data for individual degradation pathways. Importantly, literature examples demonstrate that some thermodynamically favourable degradation reactions, such as disproportionation and dimerization, can be electrochemically reversible.

Overall, this study establishes a thermodynamic framework for understanding and screening degradation mechanisms in quinone-based systems. Future work should therefore focus on integrating reversibility, kinetic modelling, explicit solvent effects, and multi-functional group interactions, as well as developing descriptors that capture dynamic stability under operating conditions.

## Supplementary Material

Supplemental Material
