# Supplementary material for: A data-driven elucidation of the challenges in designing stable quinone derivatives for aqueous organic redox flow batteries: correlations between thermodynamics of chemical degradation and energy capacity metrics
Source: Sci Technol Adv Mater. 2026 Jul 16;27(1):2698227. doi: 10.1080/14686996.2026.2698227 (PMC13439778; doi:10.1080/14686996.2026.2698227)
Supplement: Supplementary file 1 [file TSTA_A_2698227_SM9376.pdf]

Supplementary information

A data-driven elucidation of the challenges in designing stable quinone derivatives for aqueous organic redox flow batteries: correlations between thermodynamics of chemical degradation and energy capacity metrics

Ning Lu[a], Shiyao Ju[a], Daniel Willimetz[a,b], Shengming Tang[a], Abhishek Khetan[a]*

[a] *MODES, The Integrated Fuel & Chemical Science Centre, RWTH Aachen University, 52062 Aachen, Germany*

[b] *Fraunhofer Institute for Algorithms and Scientific Computing SCAI, Fraunhofer-Gesellschaft, Schloss Birlinghoven 1, 53757 Sankt Augustin, Germany*

*Corresponding author: Abhishek Khetan; Email: askhetan@modes.rwth-aachen.de

Explanation of the abbreviations used in the below tables:

SMILES: simplified molecular-input line-entry system

DFT: density functional theory

R2: the coefficient of fit

RMSE: the root mean squared error

MSE: the mean squared error

MAE: the mean absolute error

RMSD: root mean square deviation

Table S1. The 35 SMIRKS templates used to represent the eight reaction categories considered in this work, comprising seven degradation reactions and the main electrochemical redox reaction.

SMIRKS1 : [O:0]=[C:1]1-[C:2]=[C:3]-[C:4](=[O:5])-[C:6]=[C:7]-1>>[O:0]-[c:1]1:[c:2]:[c:3]:[c:4](-[O:5]):[c:6]:[c:7]:1 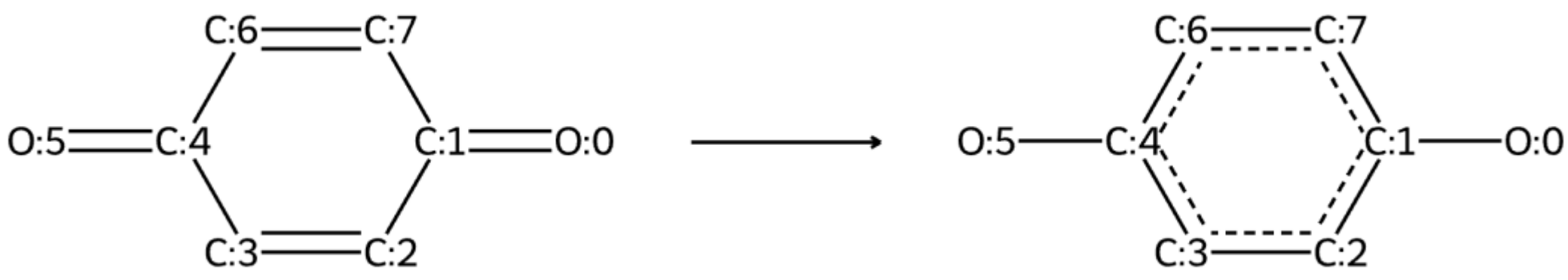
SMIRKS 2 : [O:0]=[C:1]1-[c:2]:[c:3]-[C:4](=[O:5])-[C:6]=[C:7]-1>>[O:0]-[c:1]1:[c:2]:[c:3]:[c:4](-[O:5]):[c:6]:[c:7]:1 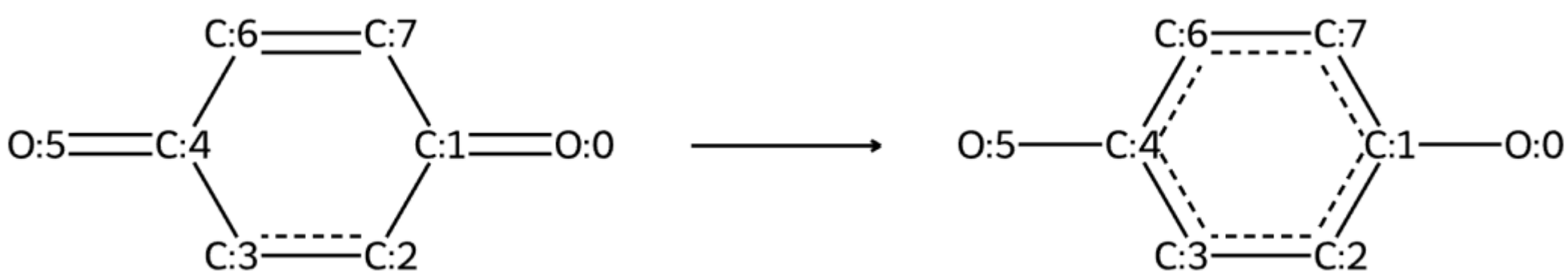
SMIRKS 3 : [O:0]=[C:1]1-[c:2]:[c:3]-[C:4](=[O:5])-[c:6]:[c:7]-1>>[O:0]-[c:1]1:[c:2]:[c:3]:[c:4](-[O:5]):[c:6]:[c:7]:1 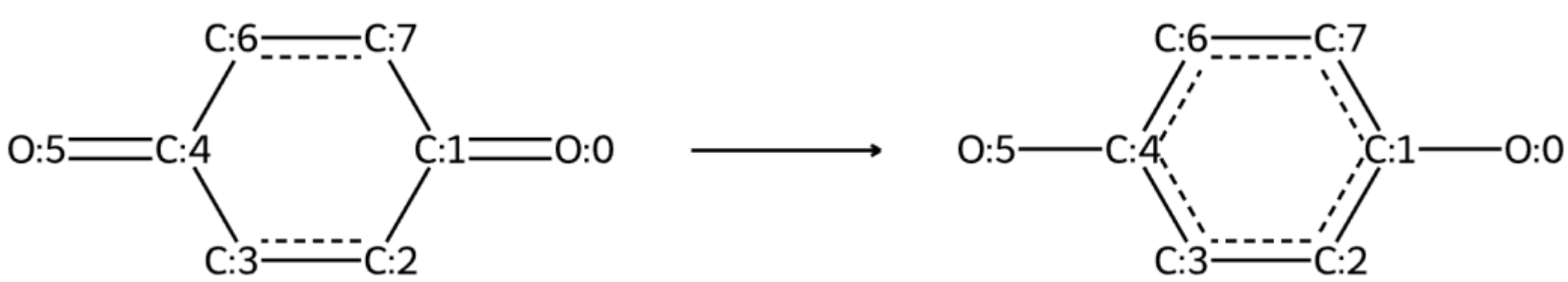

SMIRKS 4 : [O:0]=[c:1]1:[c:2]=[c:3]:[c:4](=[O:5]):[c:6]:[c:7]:1>>[O:0]-[c:1]1:[c:2]:[c:3]:[c:4](-[O:5]):[c:6]:[c:7]:1

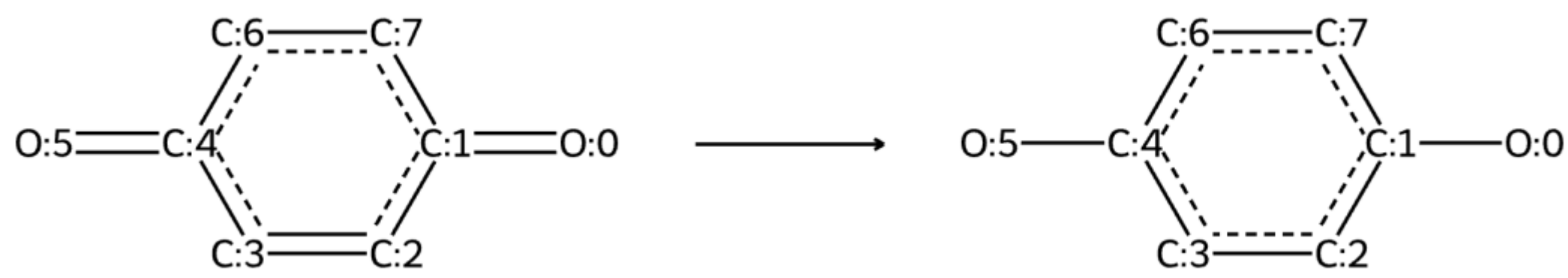

SMIRKS 5 : [O:0]=[c:1]1:[c:2]:[c:3]:[c:4](=[O:5]):[c:6]:[c:7]:1>>[O:0]-[c:1]1:[c:2]:[c:3]:[c:4](-[O:5]):[c:6]:[c:7]:1

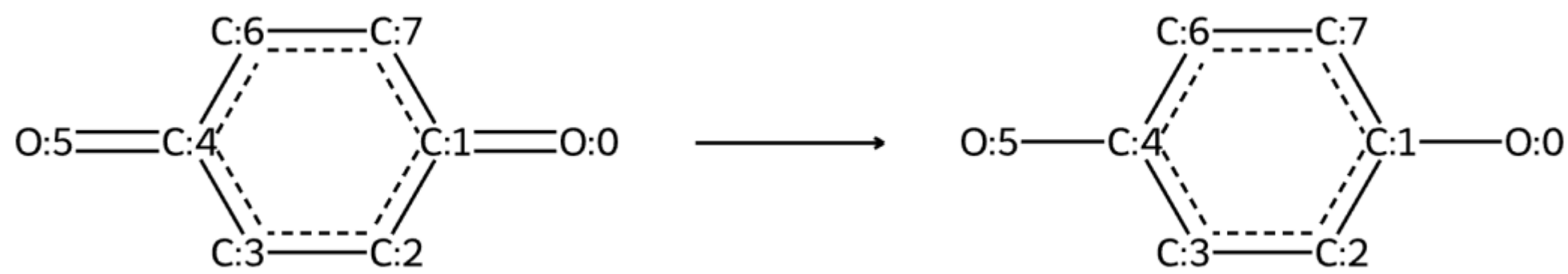

SMIRKS 6 : [O:0]=[C:1]1-[C:2]=[C:3]-[C:4](=[O:5])-[C:6]=[C:7]-1>>[O-:0]-[c:1]1:[c:2]:[c:3]:[c:4](-[O-:5]):[c:6]:[c:7]:1

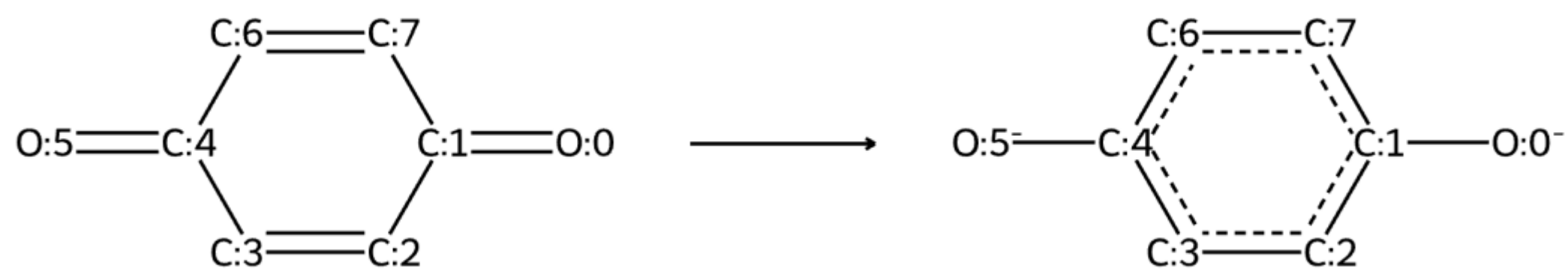

SMIRKS 7 : [O:0]=[C:1]1-[c:2]:[c:3]-[C:4](=[O:5])-[C:6]=[C:7]-1>>[O-:0]-[c:1]1:[c:2]:[c:3]:[c:4](-[O-:5]):[c:6]:[c:7]:1

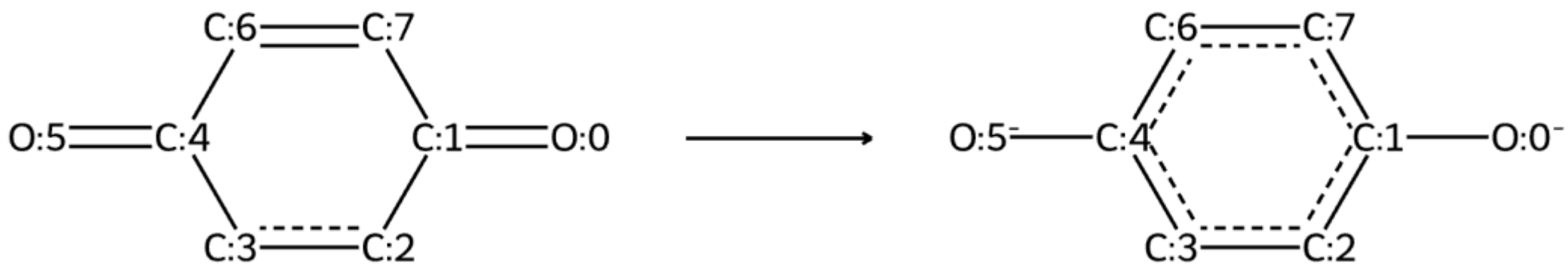

SMIRKS 8 : [O:0]=[C:1]1-[c:2]:[c:3]-[C:4](=[O:5])-[c:6]:[c:7]-1>>[O-:0]-[c:1]1:[c:2]:[c:3]:[c:4](-[O-:5]):[c:6]:[c:7]:1

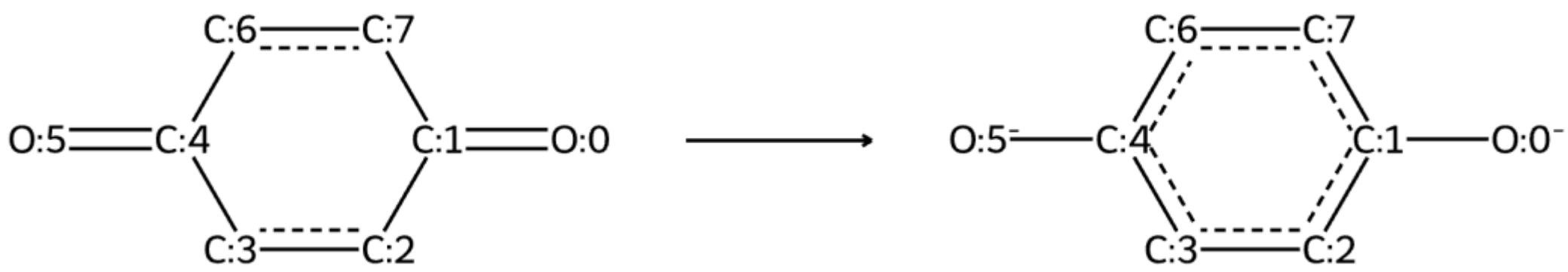

SMIRKS 9 : [O:0]=[c:1]1:[c:2]=[c:3]:[c:4](=[O:5]):[c:6]:[c:7]:1>>[O-:0]-[c:1]1:[c:2]:[c:3]:[c:4](-[O-:5]):[c:6]:[c:7]:1

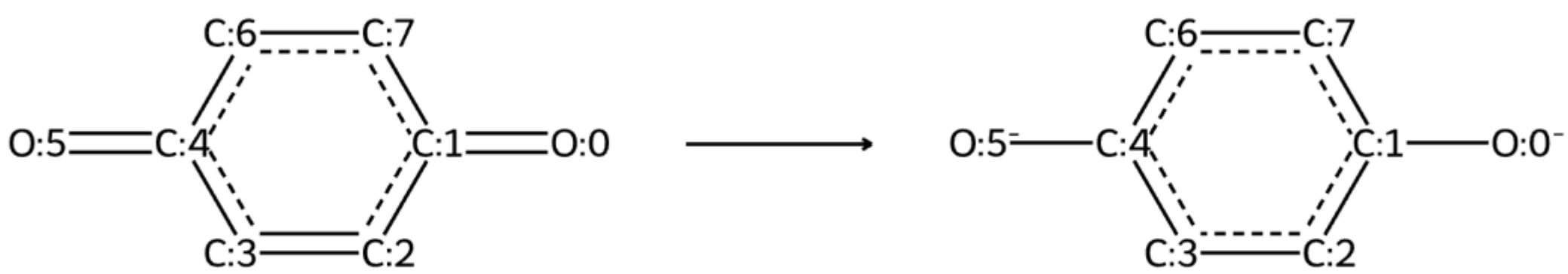

SMIRKS 10 : [O:0]=[c:1]1:[c:2]:[c:3]:[c:4](=[O:5]):[c:6]:[c:7]:1>>[O-:0]-[c:1]1:[c:2]:[c:3]:[c:4](-[O-:5]):[c:6]:[c:7]:1 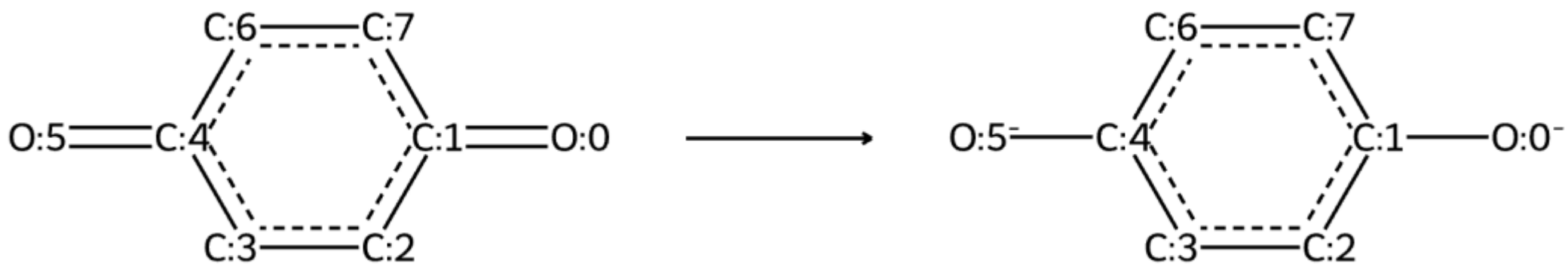
SMIRKS 11 : [O:0]=[C:1]1-[C:2]=[C:3]-[C:4](=[O:5])-[C:6]=[C:7]-1.[O;H2;+0:8]>>[O:0]=[C:1]1-[C:2]=[C:3]-[C:4](-[O:5])(-[O:8])-[C:6]=[C:7]-1 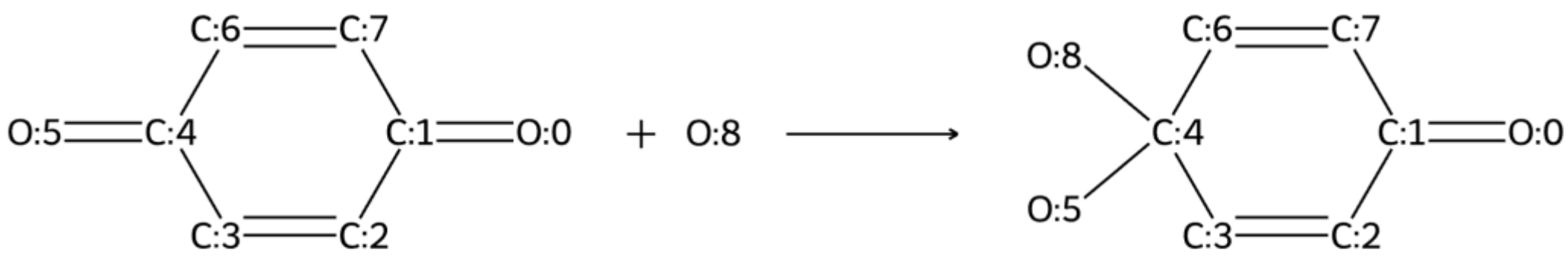
SMIRKS 12 : [O:0]=[C:1]1-[c:2]:[c:3]-[C:4](=[O:5])-[C:6]=[C:7]-1.[O;H2;+0:8]>>[O:0]=[C:1]1-[C:2]:[C:3]-[C:4](-[O:5])(-[O:8])-[C:6]=[C:7]-1 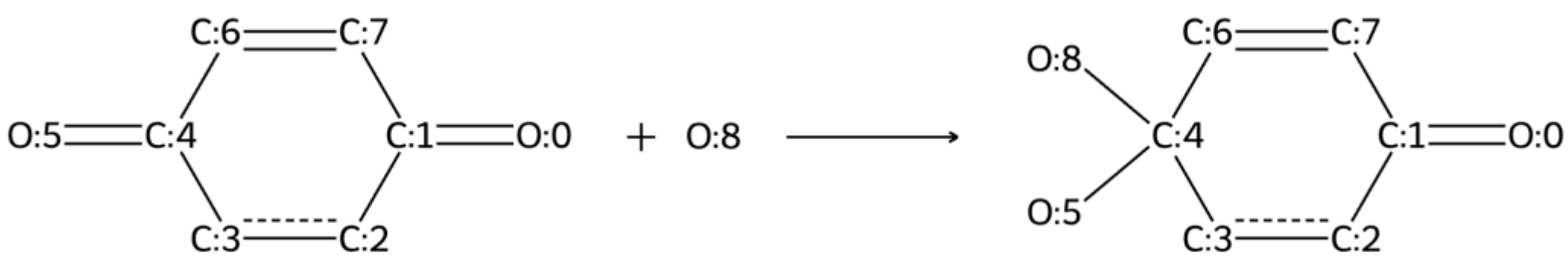

SMIRKS 13 : [O:0]=[C:1]1-[c:2]:[c:3]-[C:4](=[O:5])-[c:6]:[c:7]-1.[O;H2;+0:8]>>[O:0]=[C:1]1-[C:2]:[C:3]-[C:4](-[O:5])(-[O:8])-[C:6]:[C:7]-1

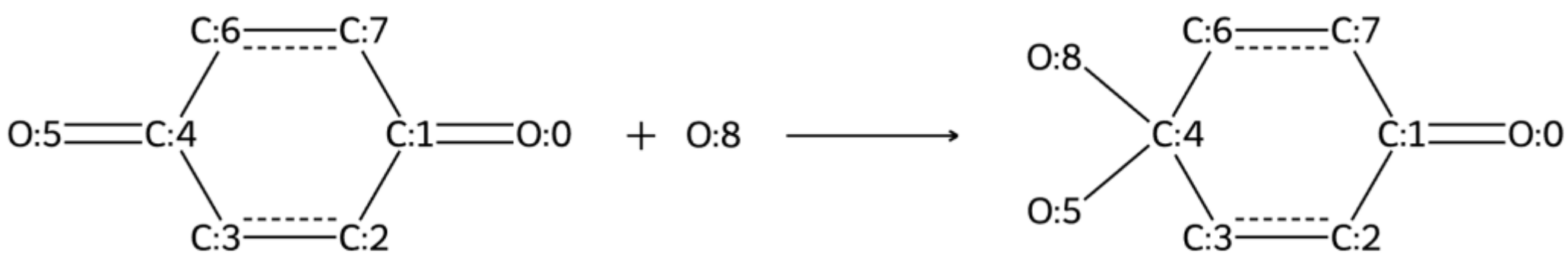

SMIRKS14 :

[O:0]=[c:1]1:[c:2]=[c:3]:[c:4](=[O:5]):[c:6]:[c:7]:1.[O;H2;+0:8]>>[O:0]=[c:1]1:[c:2]=[c:3]:[c:4](-[O:5])(-[O:8]):[c:6]:[c:7]:1

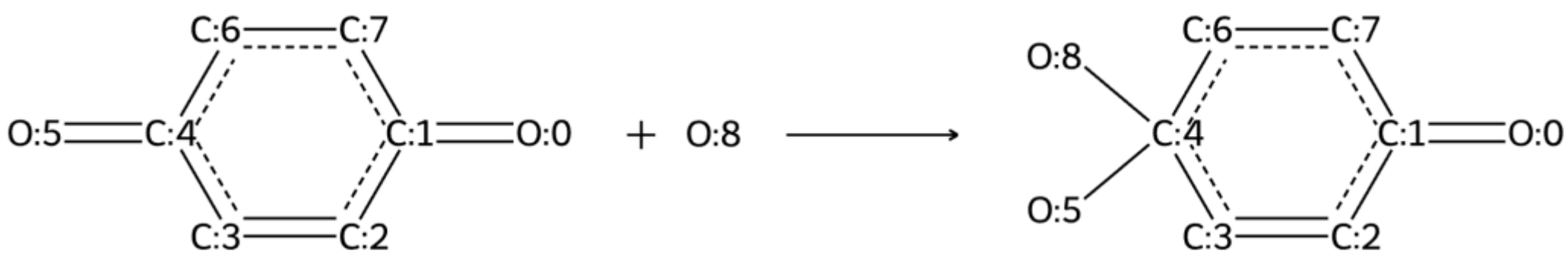

SMIRKS 15 :

[O:0]=[c:1]1:[c:2]:[c:3]:[c:4](=[O:5]):[c:6]:[c:7]:1.[O;H2;+0:8]>>[O:0]=[C:1]1-[C:2]:[C:3]-[C:4](-[O:5])(-[O:8])-[C:6]=[C:7]-1

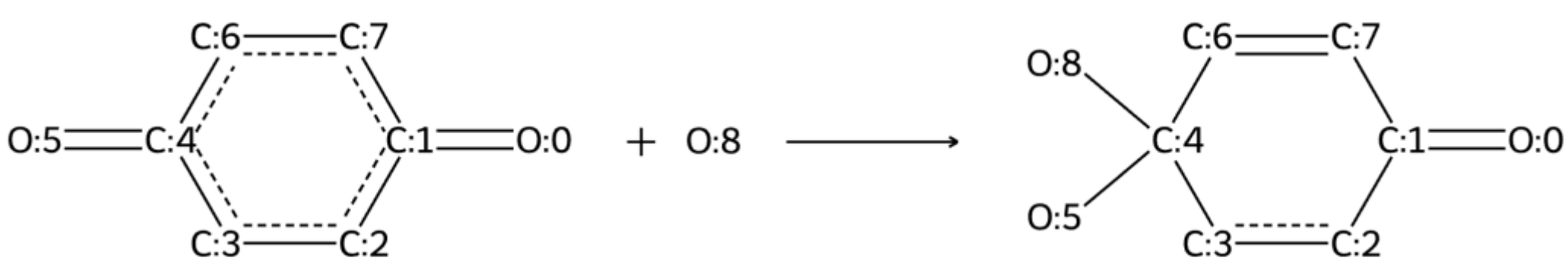

SMIRKS 16 : [C;H0;D3;+0:0]-[S:1](-[O:2])(=[O:3])=[O:4]>>[C;H0;D3;+0:0].[S:1](-[O:2])(=[O:3])=[O:4]

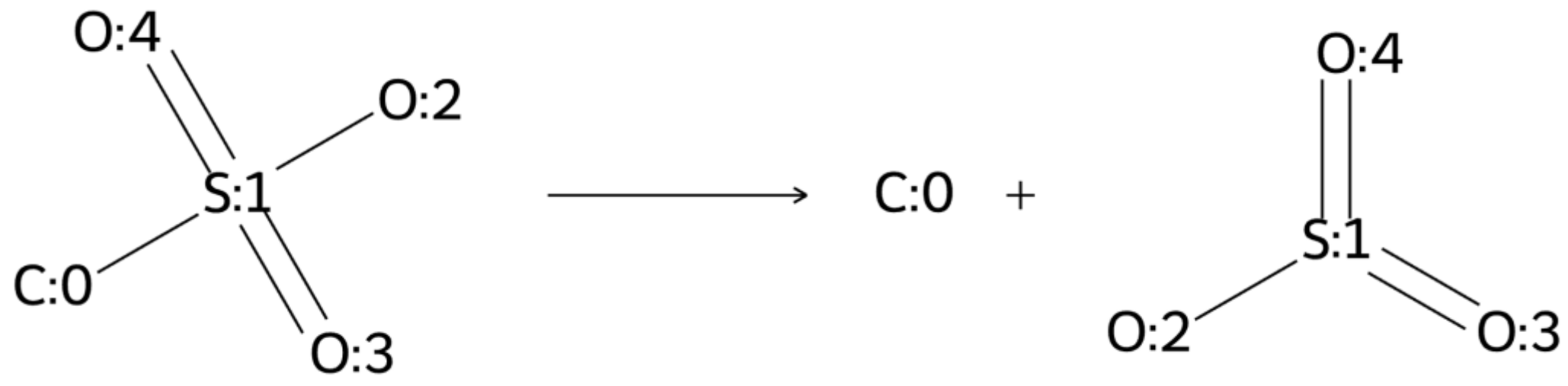

SMIRKS 17 : [c:0]-[S:1](-[O:2])(=[O:3])=[O:4]>>[c:0].[S:1](-[O:2])(=[O:3])=[O:4]

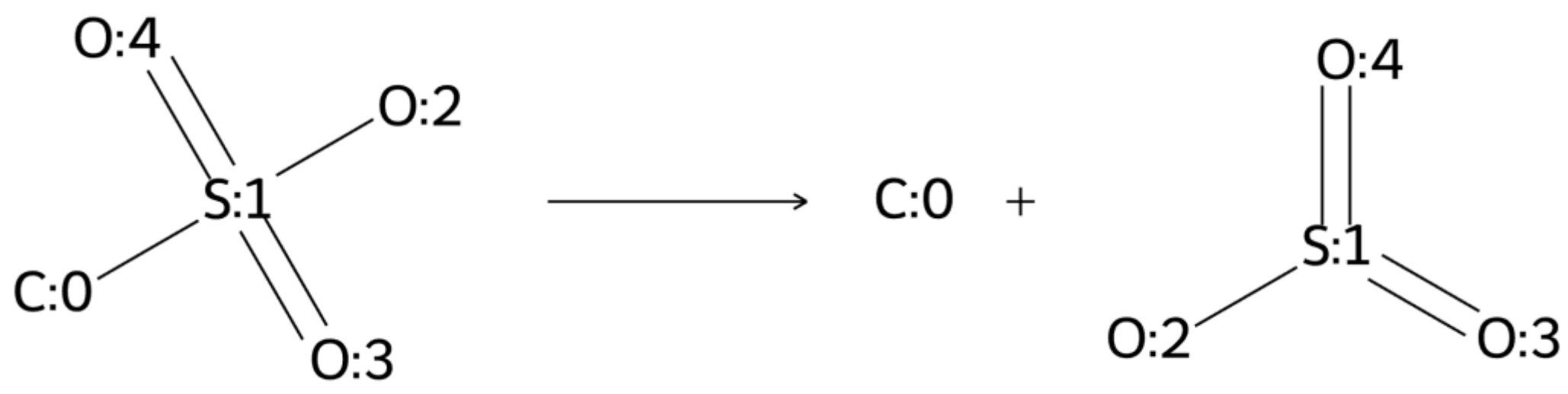

SMIRKS 18 : [O:0]=[C:1]1-[C:2]=[C:3]-[C:4](=[O:5])-[C:6]=[C;D2;+0:7]-1.[O;H2;+0:8]>>[O:0]=[C:1]1-[C:2]=[C:3]-[C:4](-[O:5])=[C:6]-[C:7]-1-[O:8]

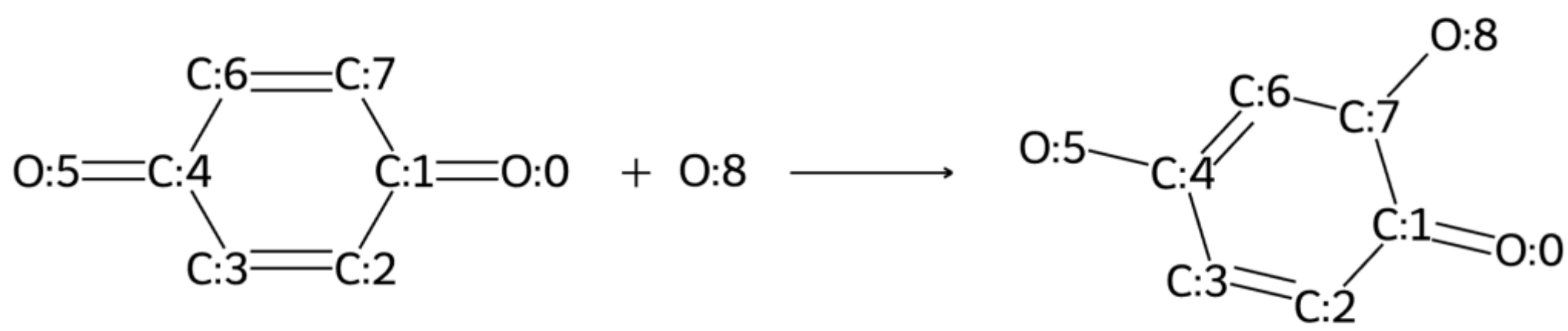

SMIRKS 19 : [O:0]=[C:1]1-[c:2]:[c:3]-[C:4](=[O:5])-[C:6]=[C;D2;+0:7]-1.[O;H2;+0:8]>>[O:0]=[C:1]1-[C:2]=[C:3]-[C:4](-[O:5])=[C:6]-[C:7]-1-[O:8]

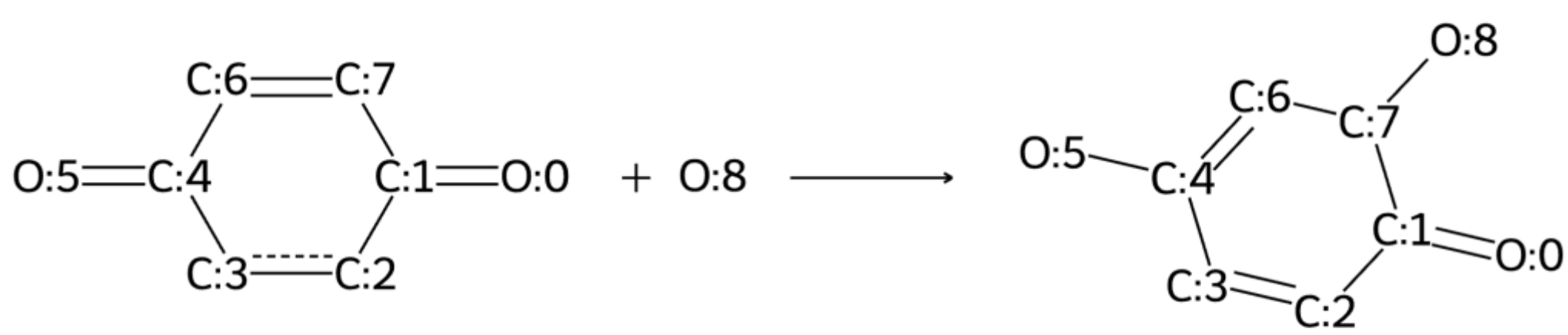

SMIRKS 20 : [O:0]=[c:1]1:[c:2]=[c:3]:[c:4](=[O:5]):[c:6]:[c;H1;D2;+0:7]:1.[O;H2;+0:8]>>[O:0]=[C:1]1-[C:2]=[C:3]-[C:4](-[O:5])=[C:6]-[C:7]-1-[O:8] 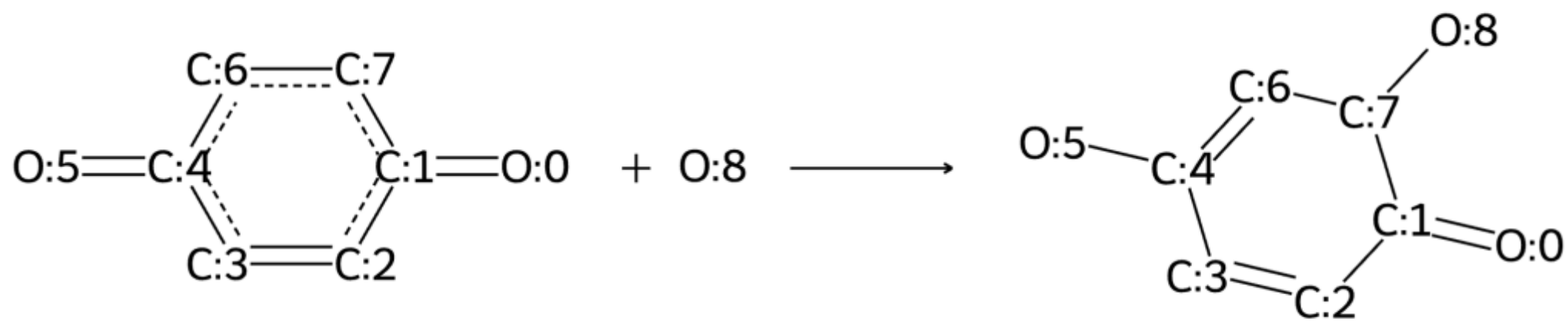
SMIRKS 21 : [O:0]=[c:1]1:[c:2]:[c:3]:[c:4](=[O:5]):[c:6]:[c;H1;D2;+0:7]:1.[O;H2;+0:8]>>[O:0]=[C:1]1-[C:2]=[C:3]-[C:4](-[O:5])=[C:6]-[C:7]-1-[O:8] 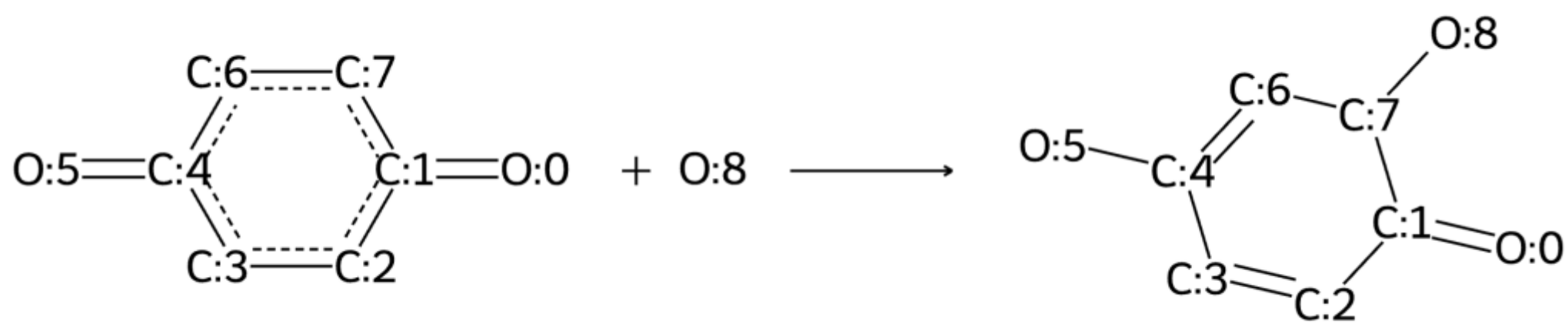
SMIRKS 22 : [C:0]-[C:1](=[O:2])-[c:3]1:[c:4]:[c:5]:[c:6]:[c:7]:[c;H1;D2;+0:8]:1.[O;H2;+0:9]>>[C:0]/[C:1](-[O:2])=[C:3]1\[C:4]=[C:5]-[C:6]=[C:7]-[C:8]-1-[O:9] 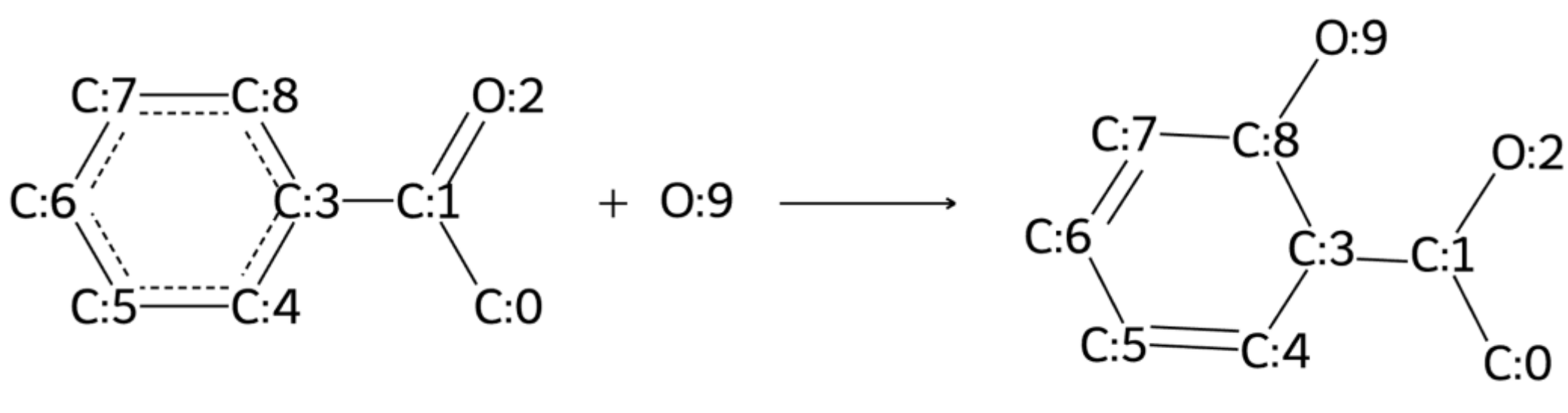
SMIRKS 23 : [c:0]-[C:1](=[O:2])-[c:3]1:[c:4]:[c:5]:[c:6]:[c:7]:[c;H1;D2;+0:8]:1.[O;H2;+0:9]>>[C:0]/[C:1](-

[O:2])=[C:3]1\[C:4]=[C:5]-[C:6]=[C:7]-[C:8]-1-[O:9]

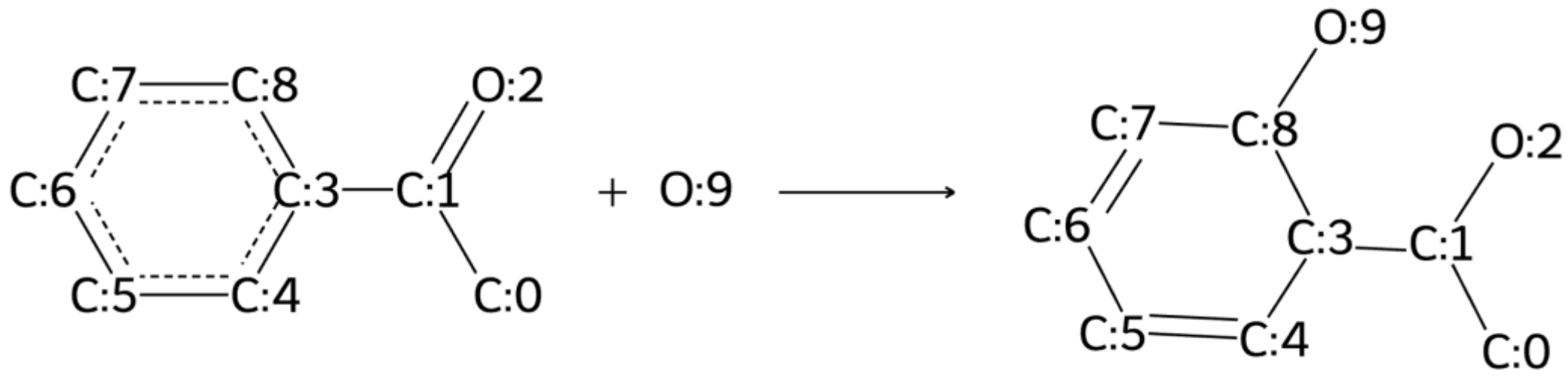

SMIRKS 24 :

[c:0]:[c:1](=[O:2]):[c:3]1:[c:4]:[c:5]:[c:6]:[c:7]:[c;H1;D2;+0:8]:1.[O;H2;+0:9]>>[C:0]/[C:1](-[O:2])=[C:3]1\[C:4]=[C:5]-[C:6]=[C:7]-[C:8]-1-[O:9]

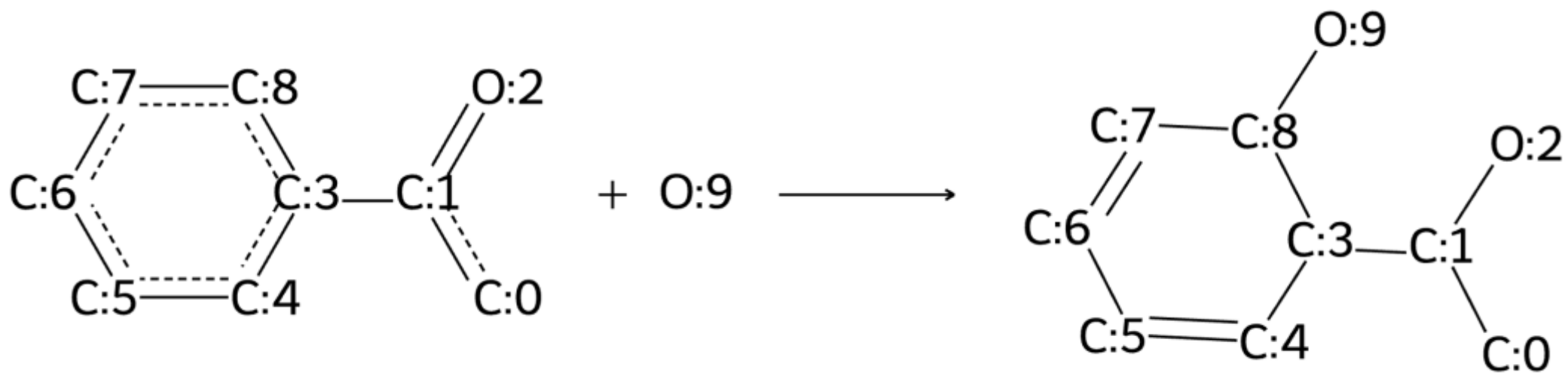

SMIRKS 25 : [O:0]=[C:1]1-[C:2]=[C:3]-[C:4](=[O:5])-[C:6]=[C:7]-1>>[O:0]=[c:1]1-[c:2]=[c:3]-[c:4]-[c:6]=[c:7]-1.[O;H2;+0:5]

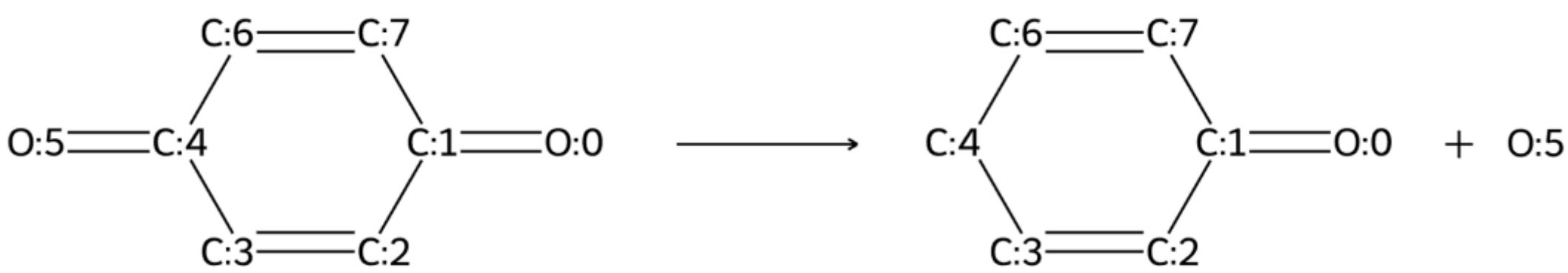

SMIRKS 26 : [O:0]=[C:1]1-[c:2]:[c:3]-[C:4](=[O:5])-[C:6]=[C:7]-1>>[O:0]=[c:1]1-[c:2]:[c:3]-[c:4]-[c:6]=[c:7]-1.[O;H2;+0:5]

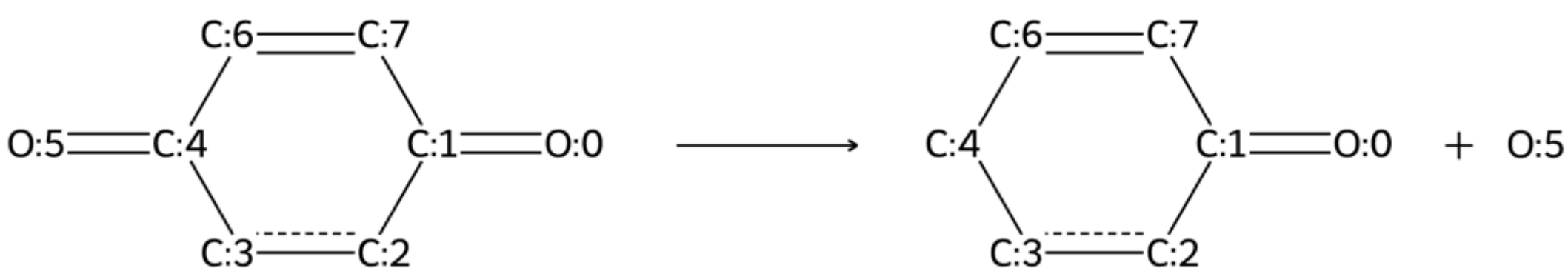

SMIRKS 27 : [O:0]=[C:1]1-[c:2]:[c:3]-[C:4](=[O:5])-[c:6]:[c:7]-1>>[O:0]=[c:1]1-[c:2]:[c:3]-[c:4]-[c:6]:[c:7]-1.[O;H2;+0:5]

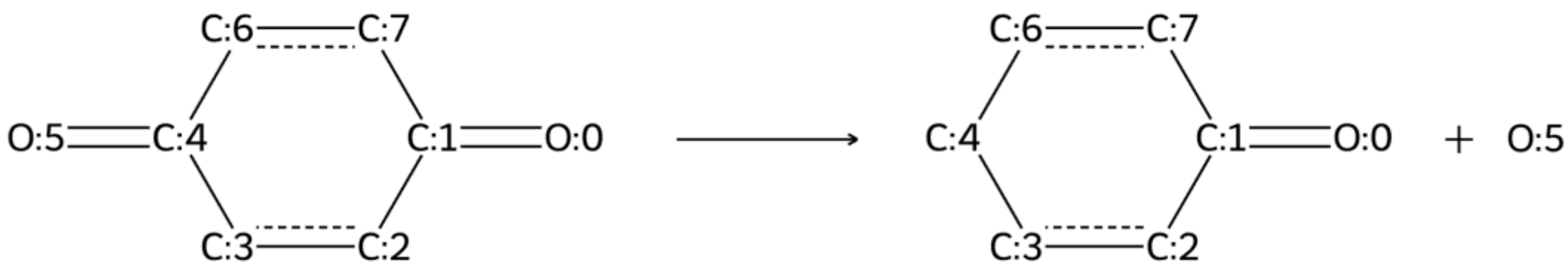

SMIRKS 28 : [O:0]=[c:1]1:[c:2]=[c:3]:[c:4](=[O:5]):[c:6]:[c:7]:1>>[O:0]=[C:1]1-[c:2]:[c:3]-[C:4]-[C:6]=[C:7]-1.[O;H2;+0:5]

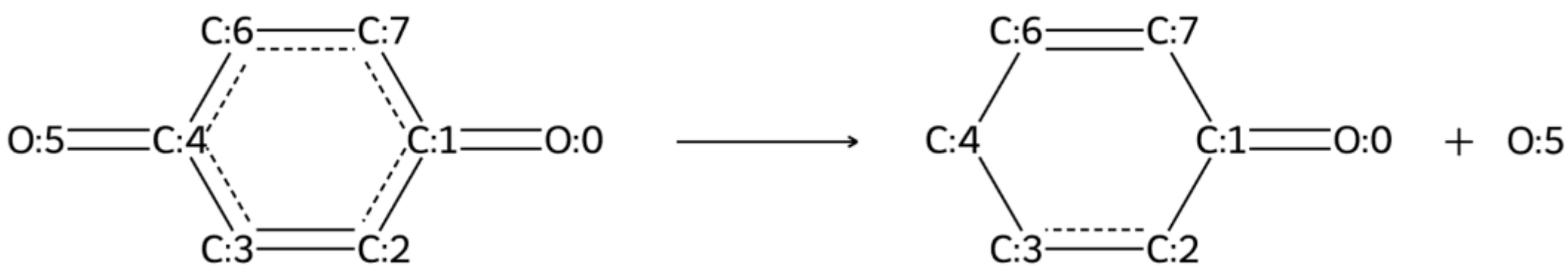

SMIRKS 29 : [O:0]=[c:1]1:[c:2]:[c:3]:[c:4](=[O:5]):[c:6]:[c:7]:1>>[O:0]=[C:1]1-[c:2]:[c:3]-[C:4]-[C:6]=[C:7]-1.[O;H2;+0:5]

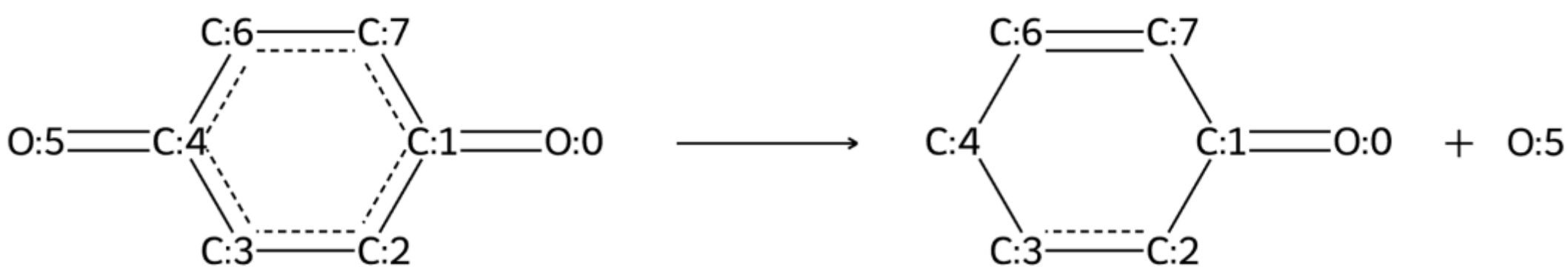

SMIRKS 30 : [O;v2;+0:0]-[c:1]1:[c:2]:[c:3]:[c:4](-[O;v2;+0:5]):[c:6]:[c:7]:1>>[O:0]-[C:1]1=[C:2]-[C:3]-[C:4](=[O:5])-[C:6]=[C:7]-1

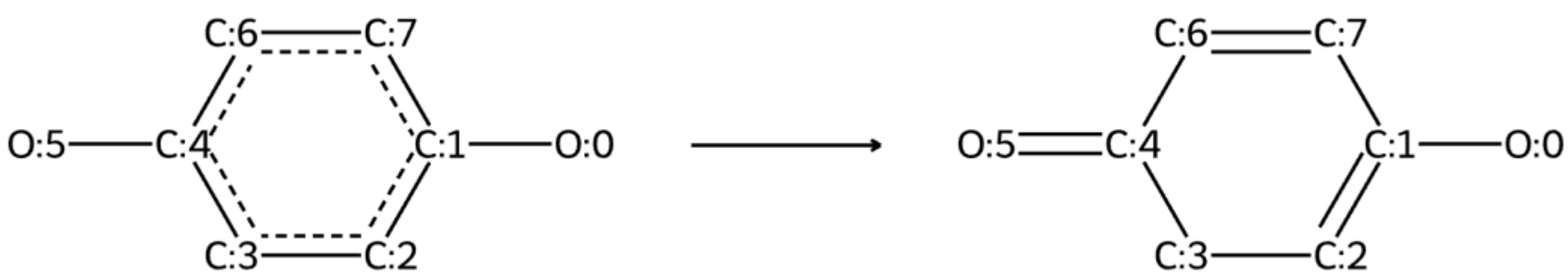

SMIRKS 31 : [O;v2;+0:0]-[c:1]1:[c;D3;+0:2]:[c;D3;+0:3]:[c:4](-[O;v2;+0:5]):[c:6]2:[c:7]:[c:8]:[c:9]:[c:10]:[c:11]:1:2>>[O:0]-[C:1]1-[C:2]=[C:3]-[C:4](=[O:5])-[C:6]2-[C:7]=[C:8]-[C:9]=[C:10]-[C:11]=1-2

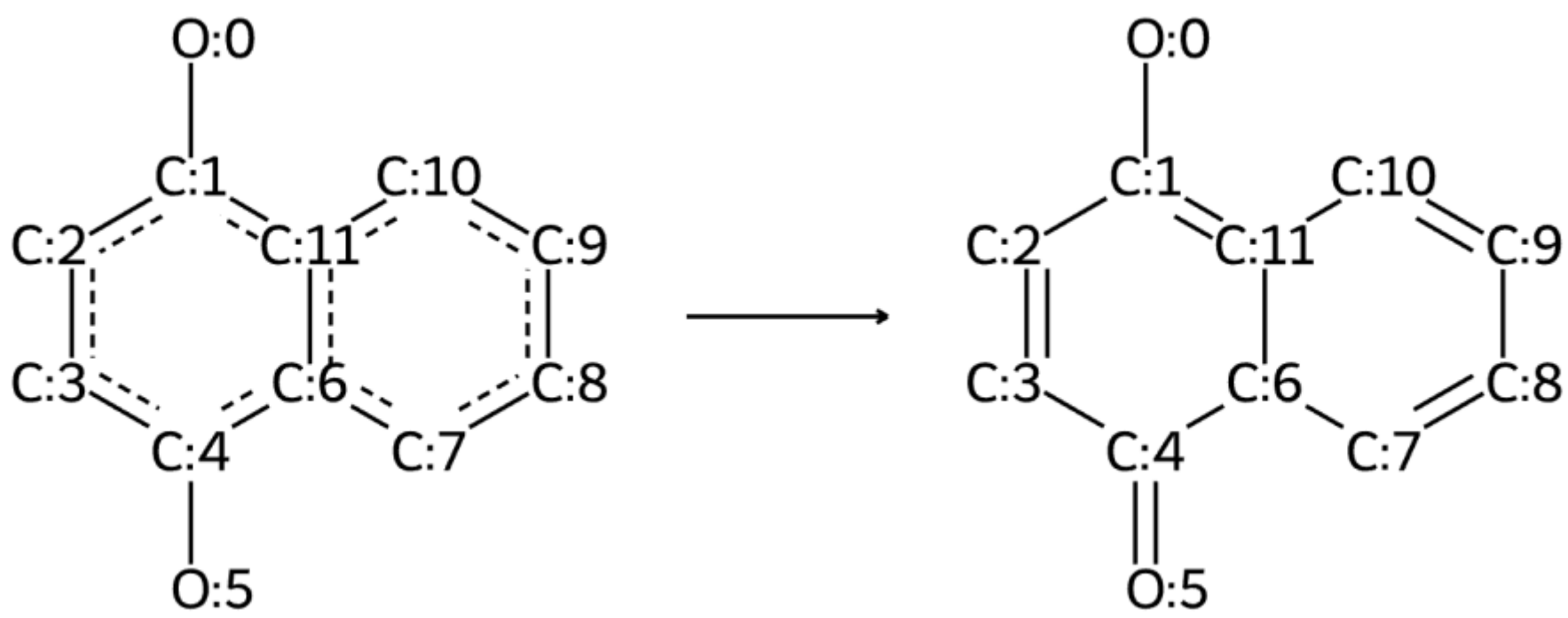

SMIRKS 32 : [O:0]=[C:1]1-[C:2]=[C:3]-[C:4](-[O:5])=[C:6]-[C:7]-1>>[O:0]=[C:1]1-[C:2]=[C:3]-[C:4](=[O:5])-[C:6]-[C:7]-1

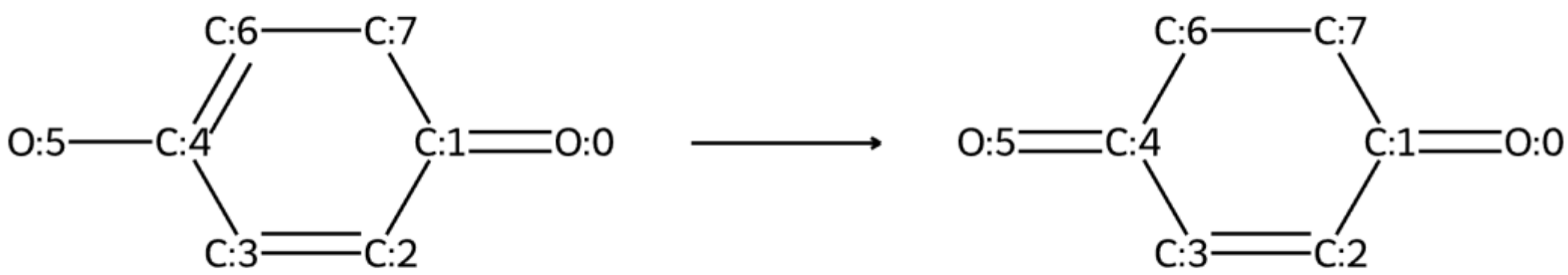

SMIRKS 33 : [O:0]=[C:1]1-[c:2]:[c:3]-[C:4](-[O:5])=[C:6]-[C:7]-1>>[O:0]=[C:1]1-[C:2]=[C:3]-[C:4](=[O:5])-[C:6]-[C:7]-1

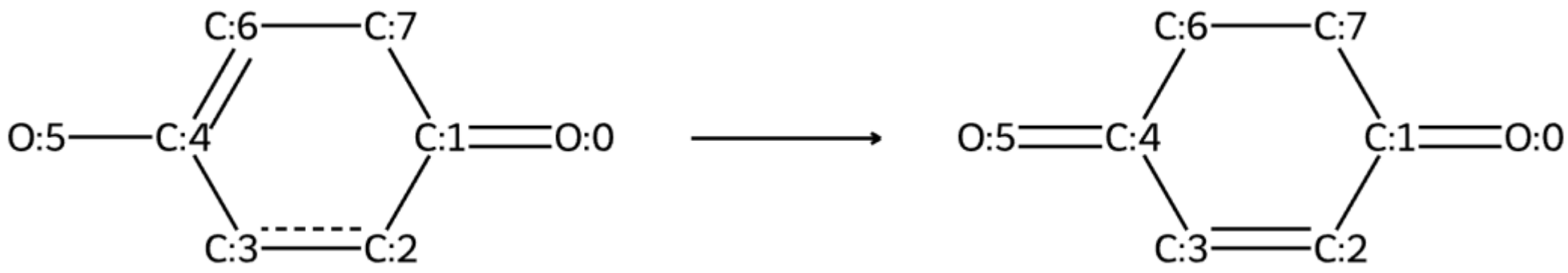

SMIRKS 34 : [O-:0]-[c:1]1:[c:2]:[c:3]:[c:4](-[O-:5]):[c:6]:[c:7]:1>>[O;H0;+0:0]=[c:1]1-[c:2]=[c:3]-[c-:4]-[c:6]=[c:7]-1.[O;H2;+0:5]

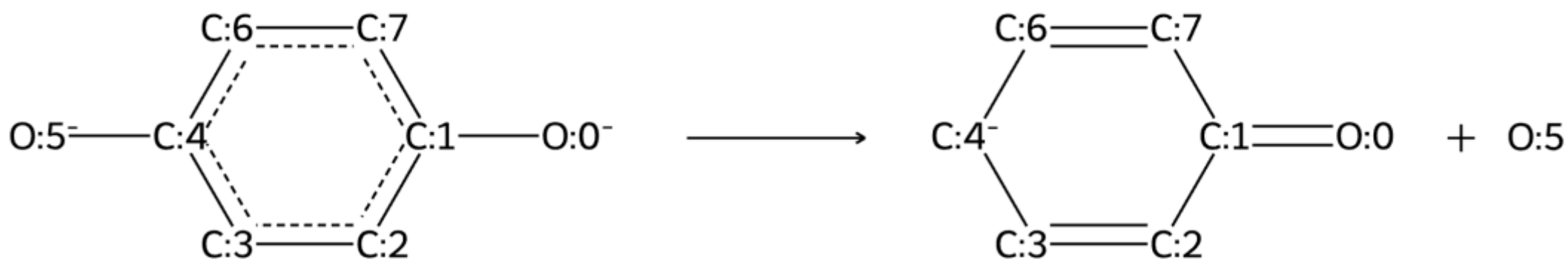

SMIRKS 35 : [O:0]=[c:1]1:[c:2]:[c:3]:[cH-:4]:[c:5]:[c:6]:1.[O:7]=[c:8]1:[c:9]:[c:10]:[cH-:11]:[c:12]:[c:13]:1>>[O:0]=[C:1]1-[C:2]=[C:3]-[C;H1;+0:4](-[C;H1;+0:11]2-[C:12]=[C:13]-[C:8](=[O:7])-[C:9]=[C:10]-2)-[C:5]=[C:6]-1

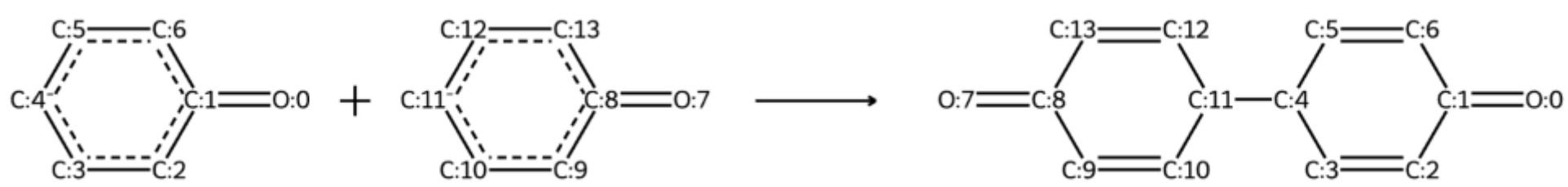

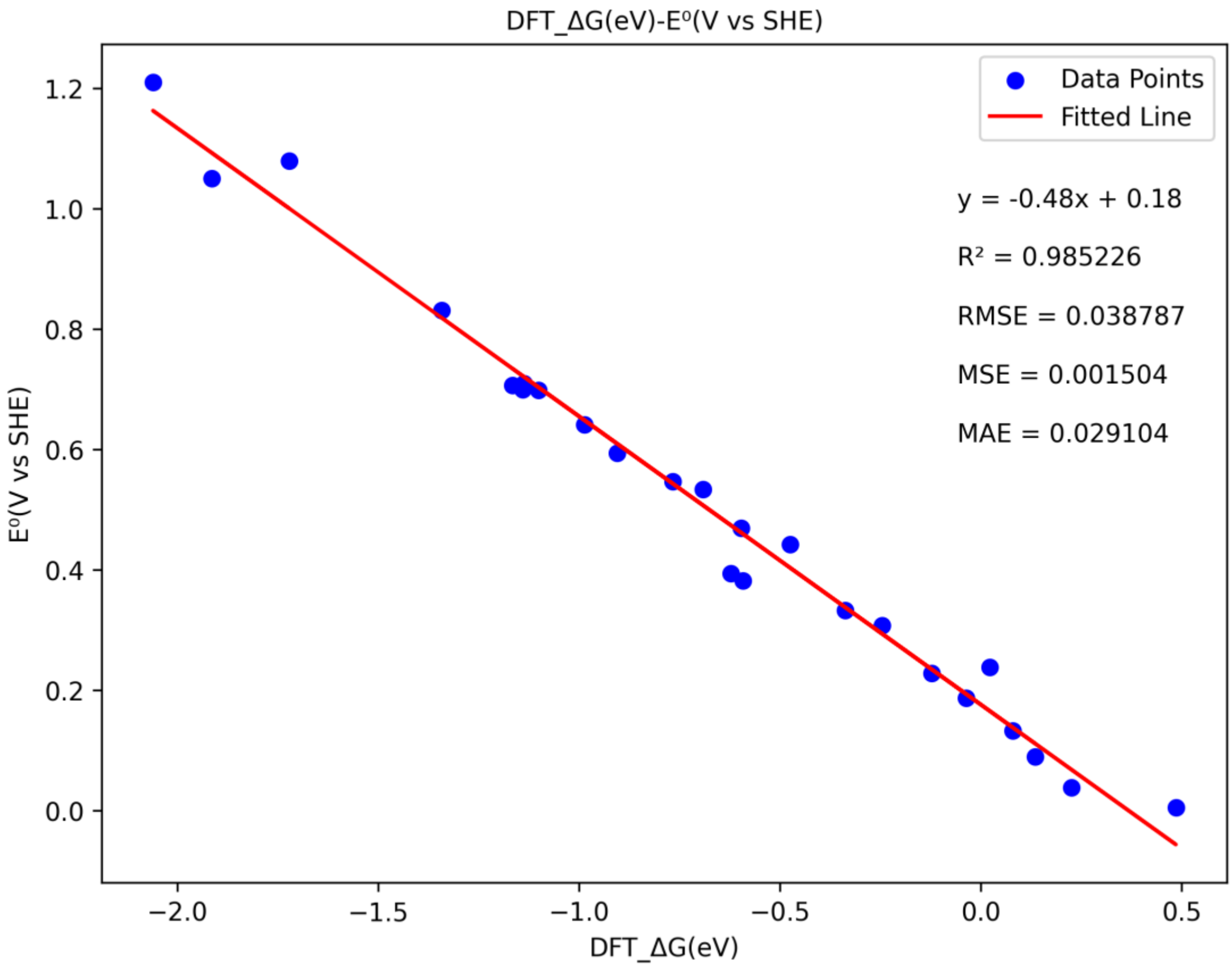

Figure S1. The simulation between the free energy and the redox potential in experiments.

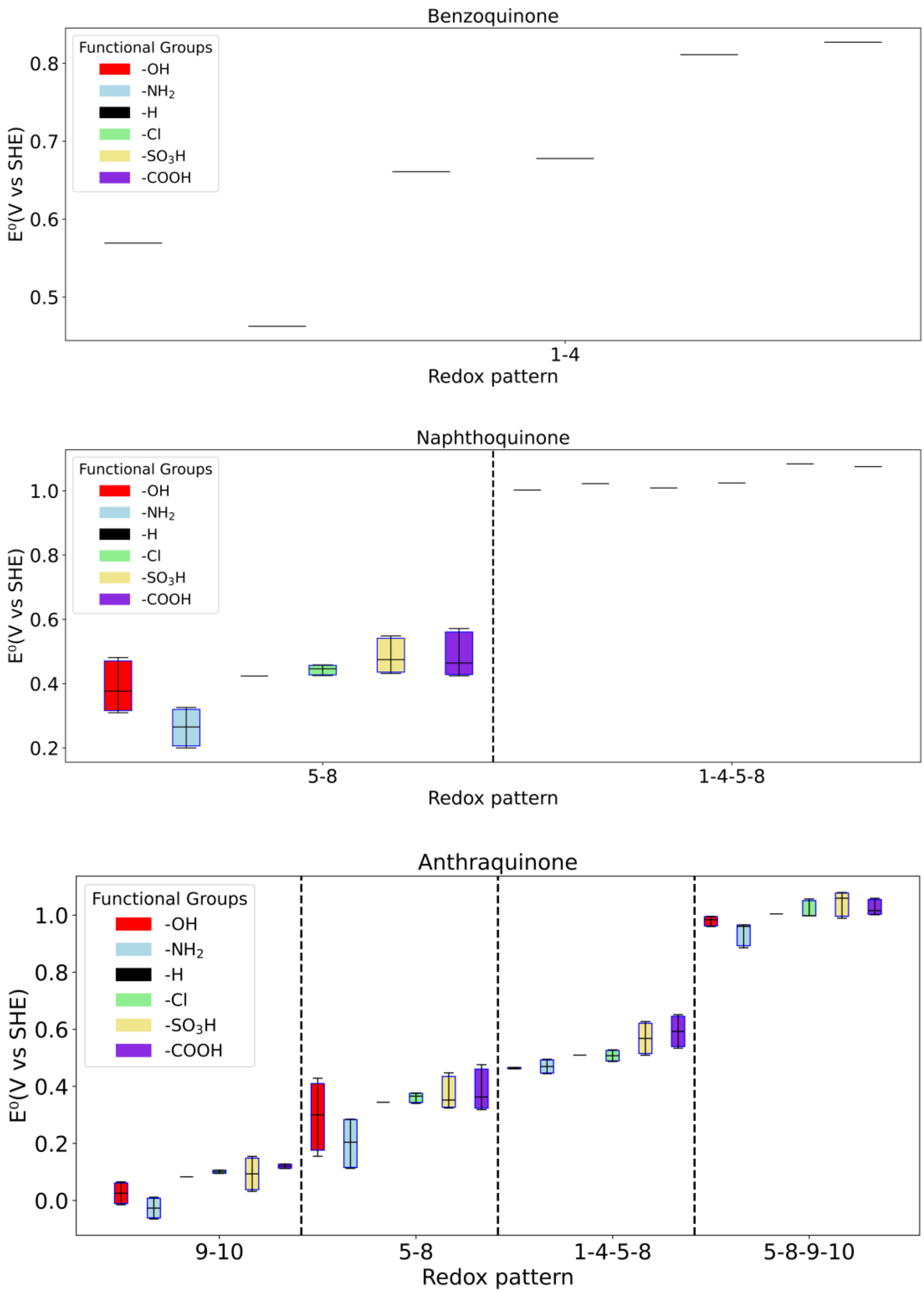

Figure S2. Redox potential property relationships. The reduction potential depends on both the redox pattern (the relative positions of the carbonyl groups) and the functional groups. The numbers refer to the molecules of the redox pattern and correspond to the labels in Fig. 1. The median of each set of data is indicated with a black line. The regions

bounded by the colours represent the range of the 5th and 95th percentiles of reduction potentials. The lines extend out to the maximum and minimum reduction potential for each substitution and ketone

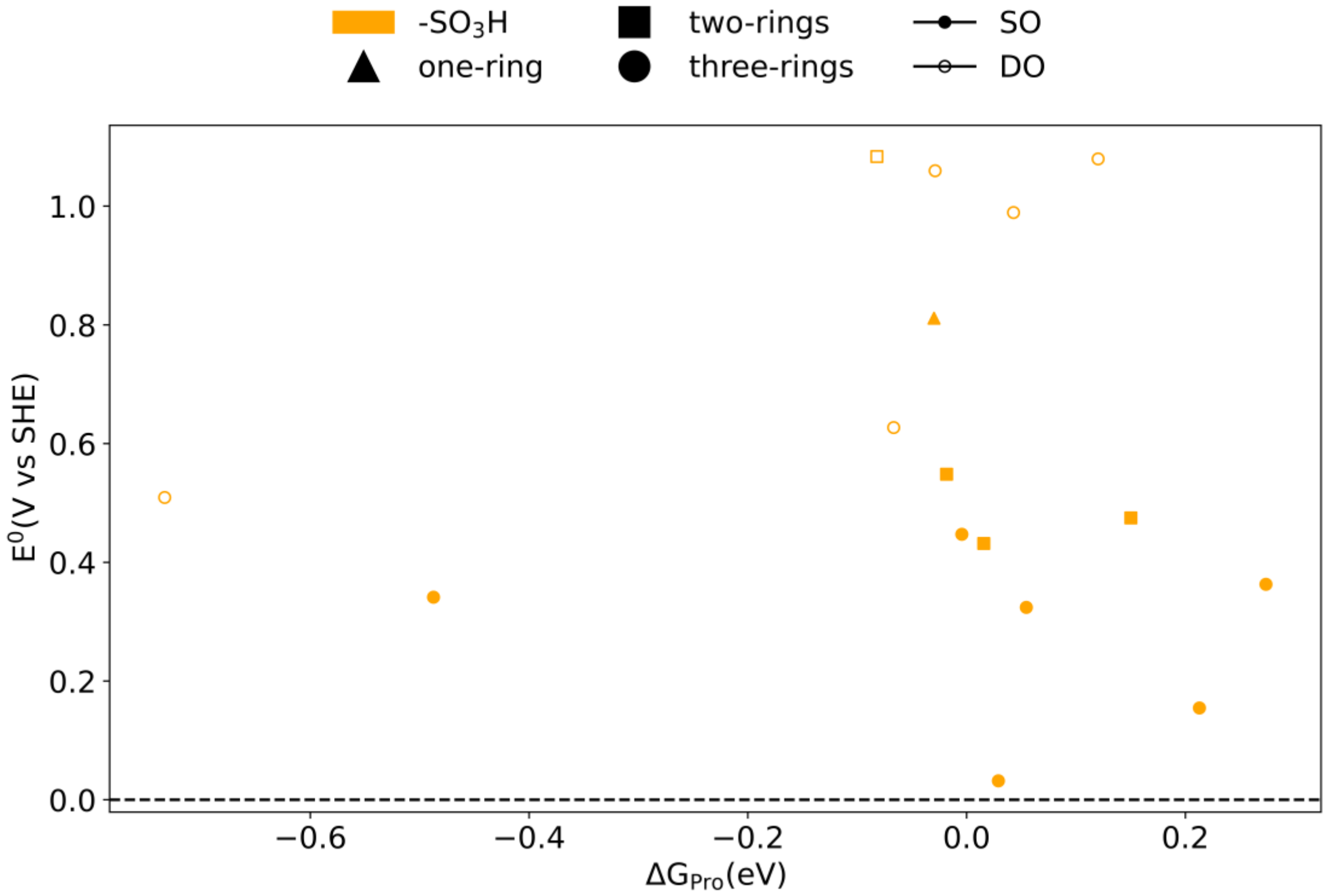

Figure S3. The relationship between the predicted redox potential and the Gibbs free energy of Proto-desulfonation.

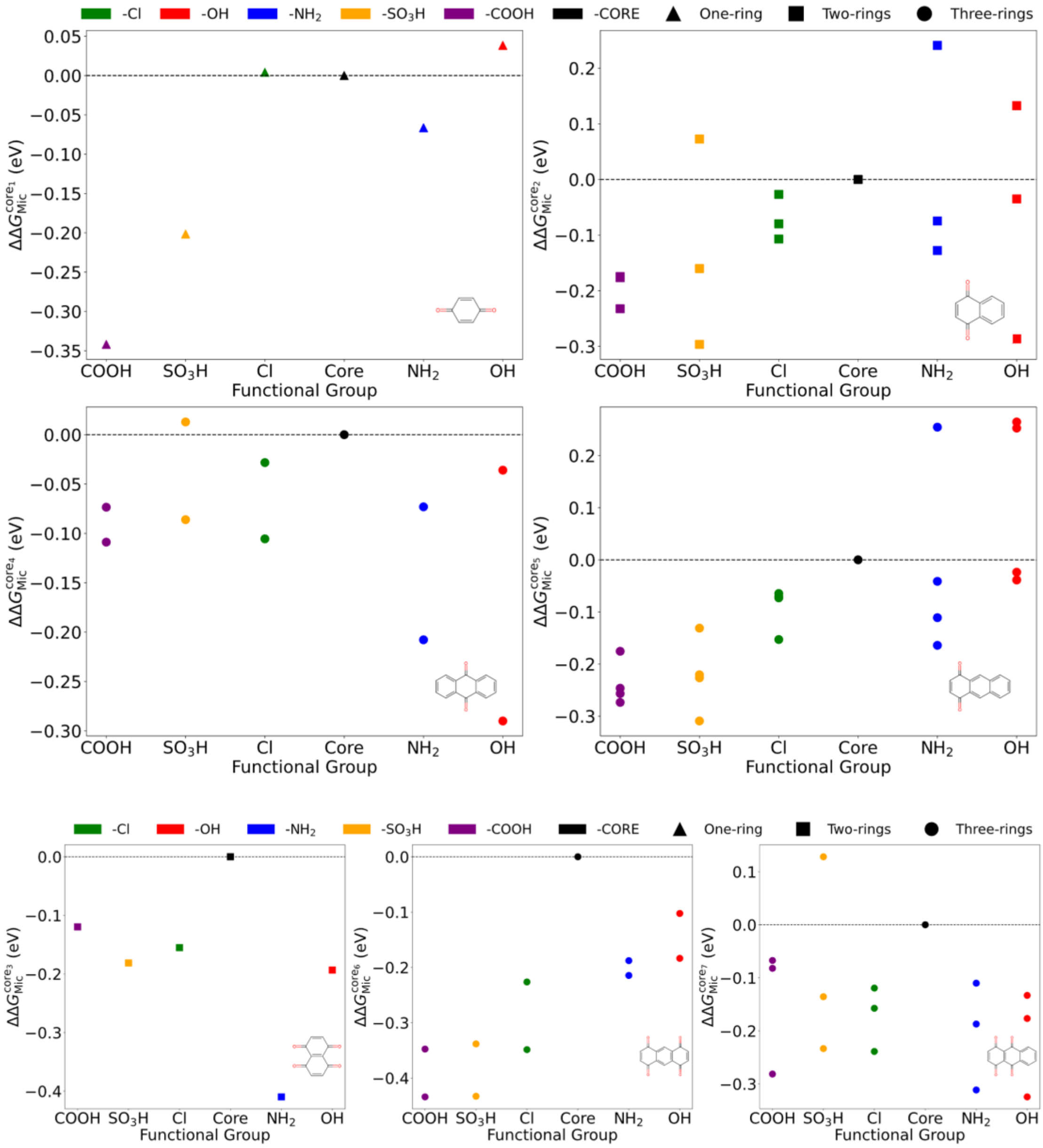

Figure S4. Effect of functional groups on stability based on core structure under Michael addition mechanisms

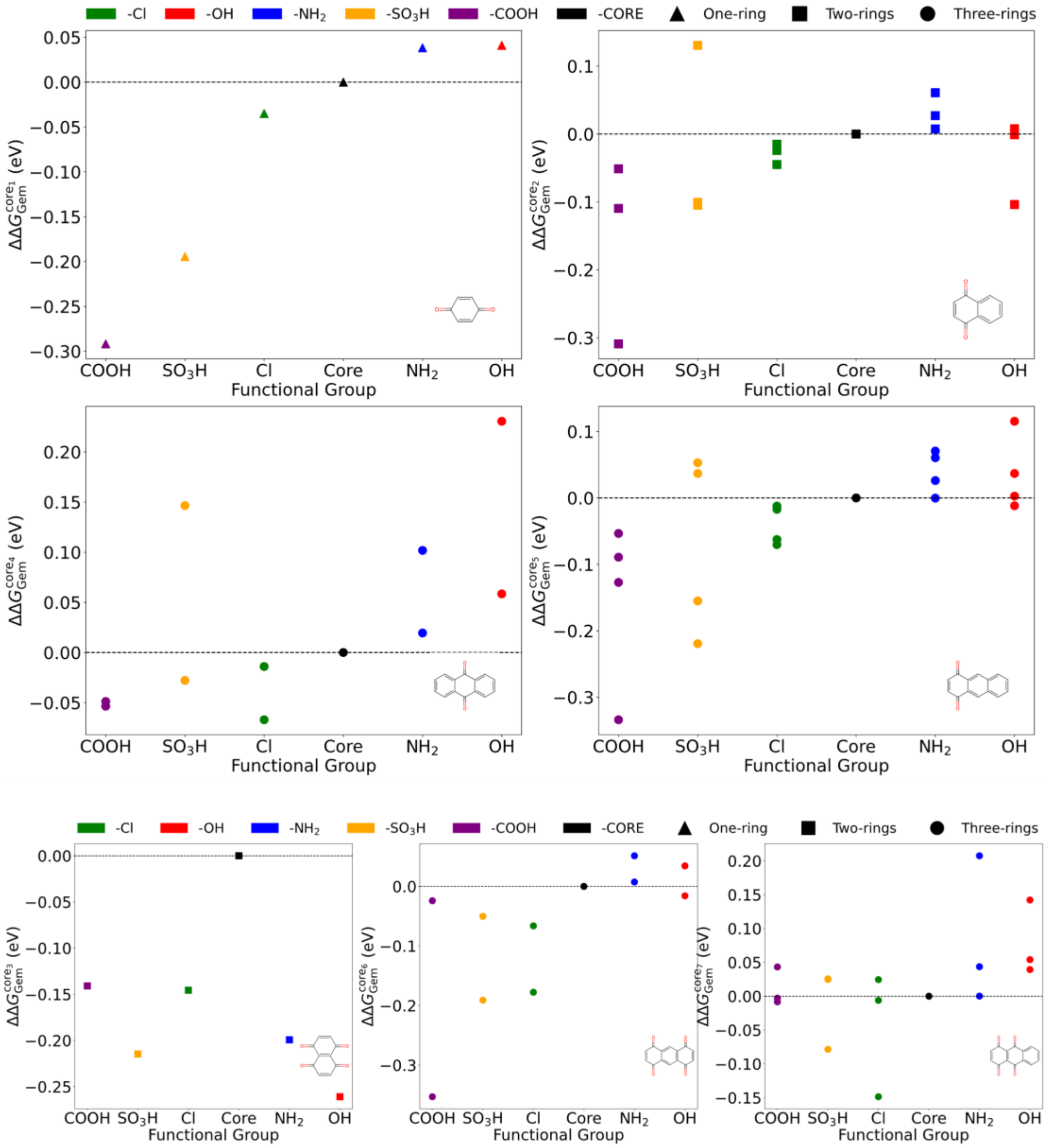

Figure S5. Effect of functional groups on stability based on core structure under Gem-Diol Formation mechanisms.

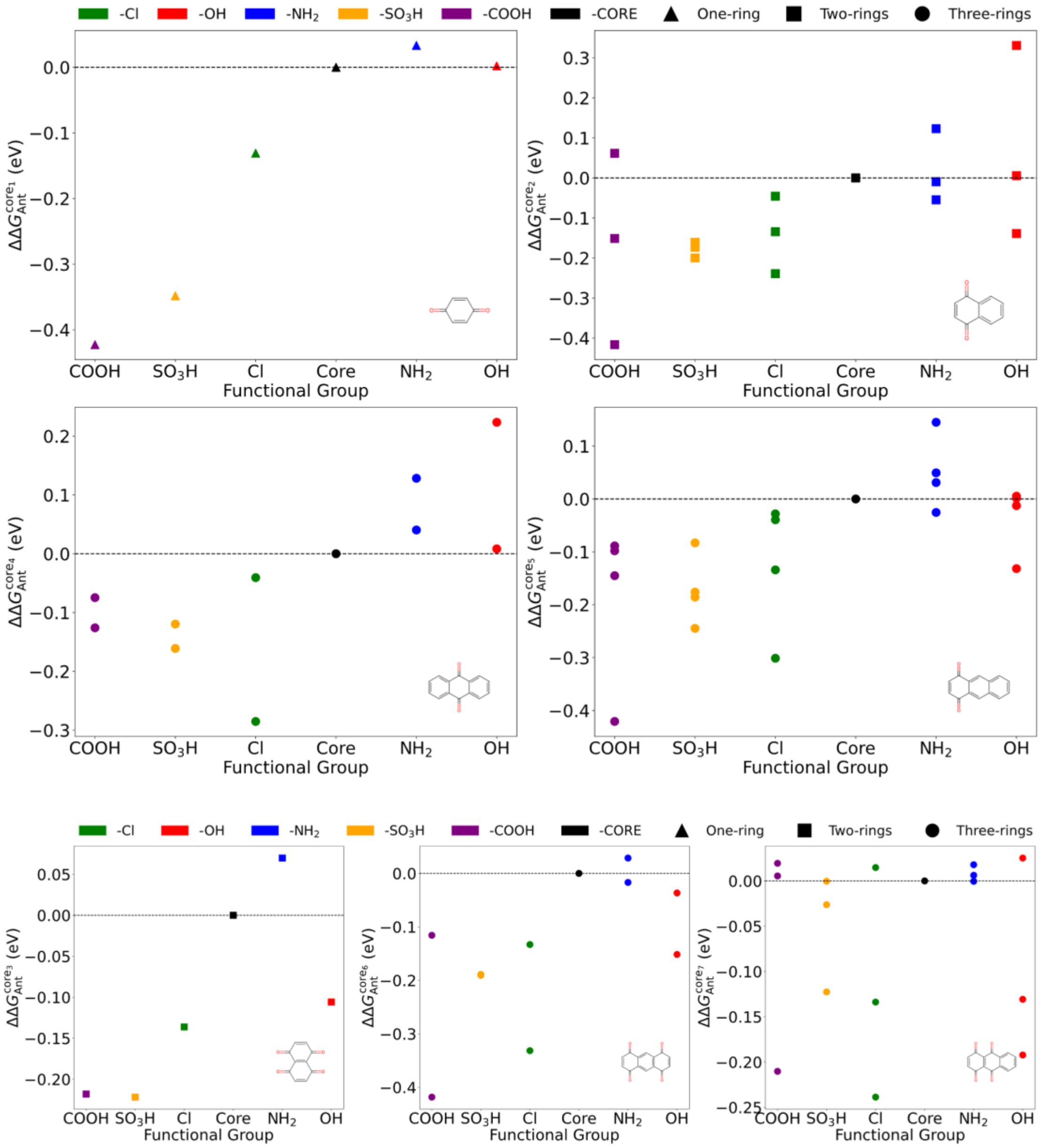

Figure S6. Effect of functional groups on stability based on core structure under the Anthrone formation mechanism.

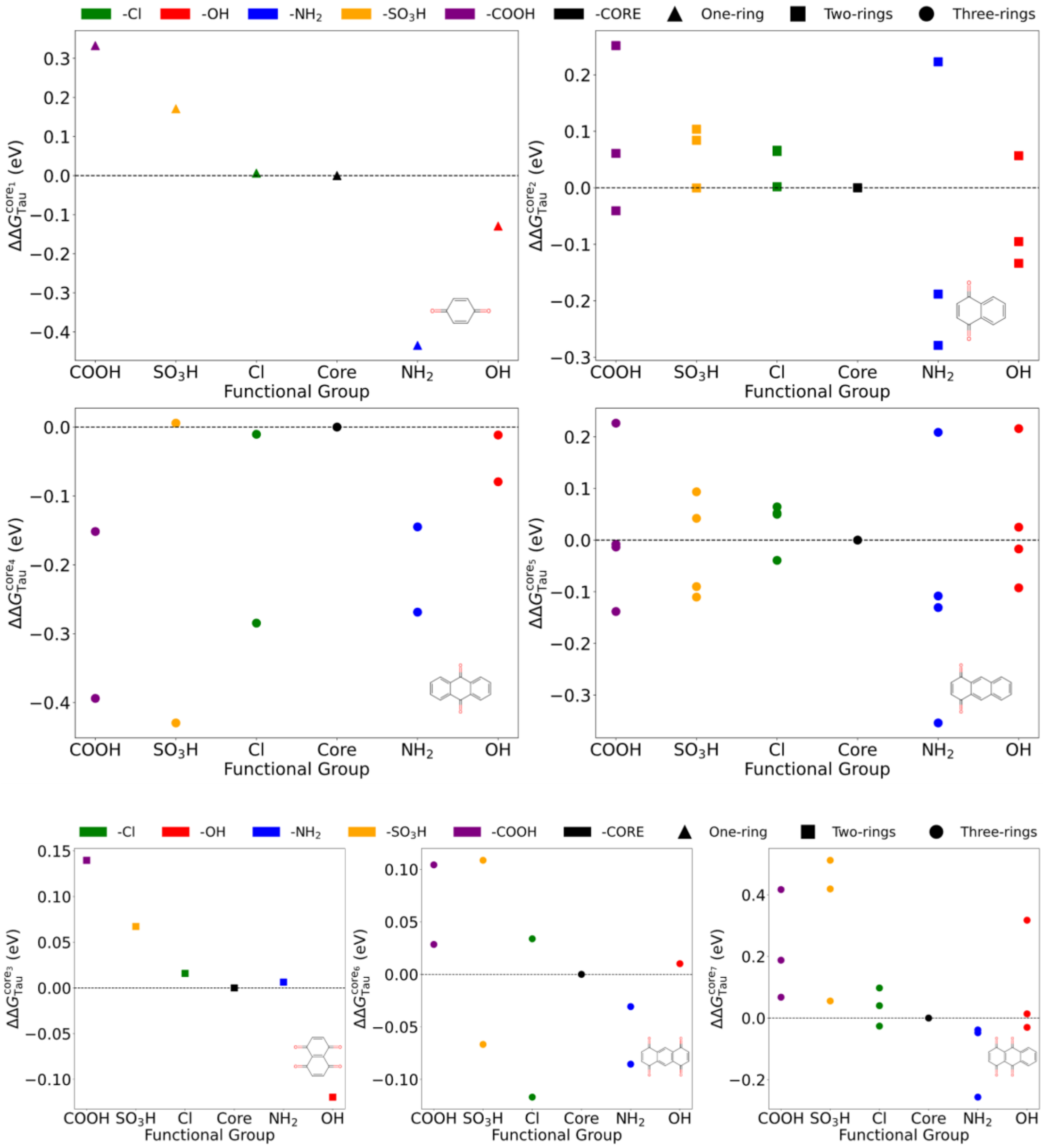

Figure S7. Effect of functional groups on stability based on core structure under the Tautomerization mechanism.

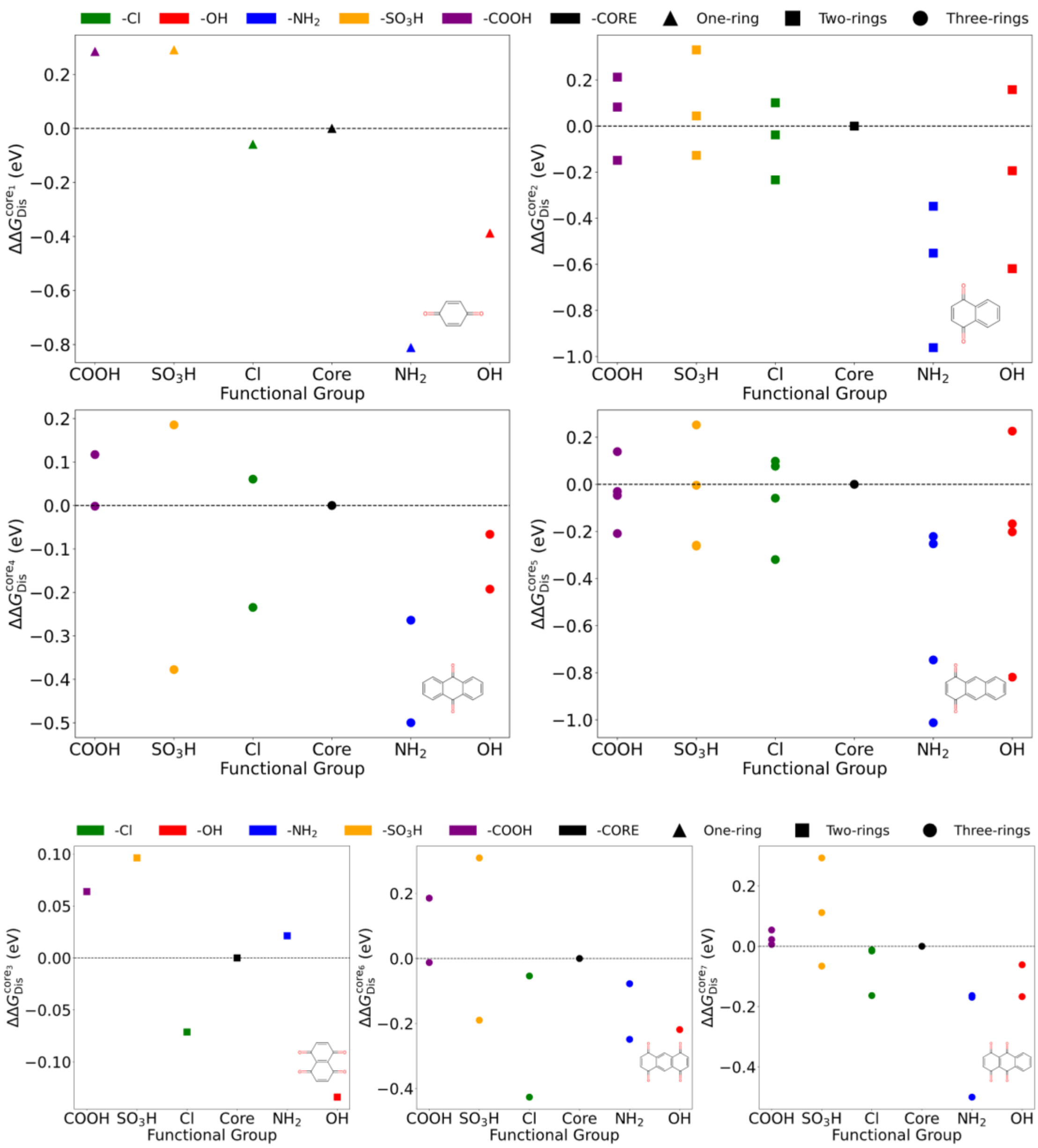

Figure S8. Effect of functional groups on stability based on core structure under the Disproportionation mechanism.

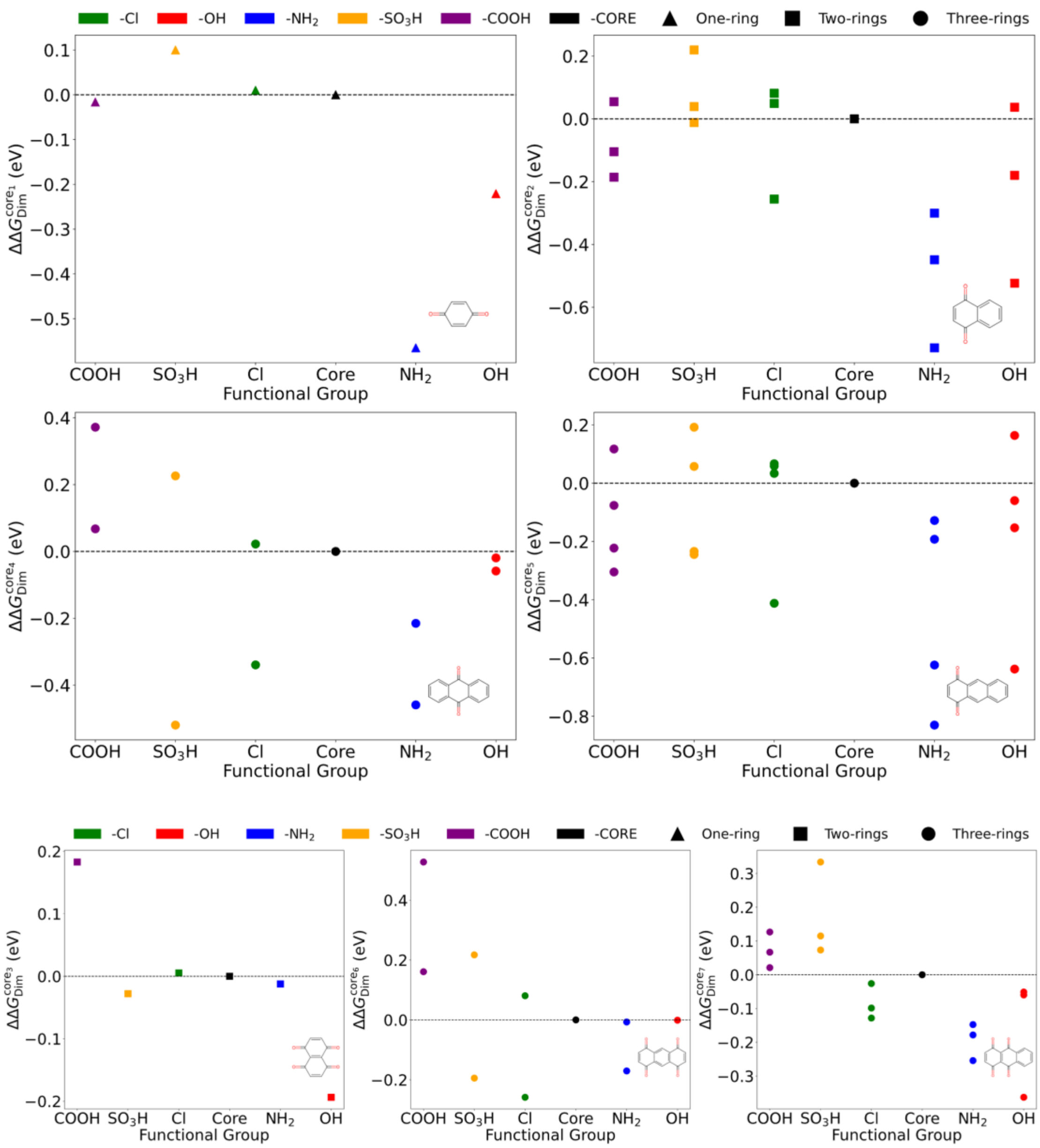

Figure S9. Effect of functional groups on stability based on core structure under the Dimerization mechanism.

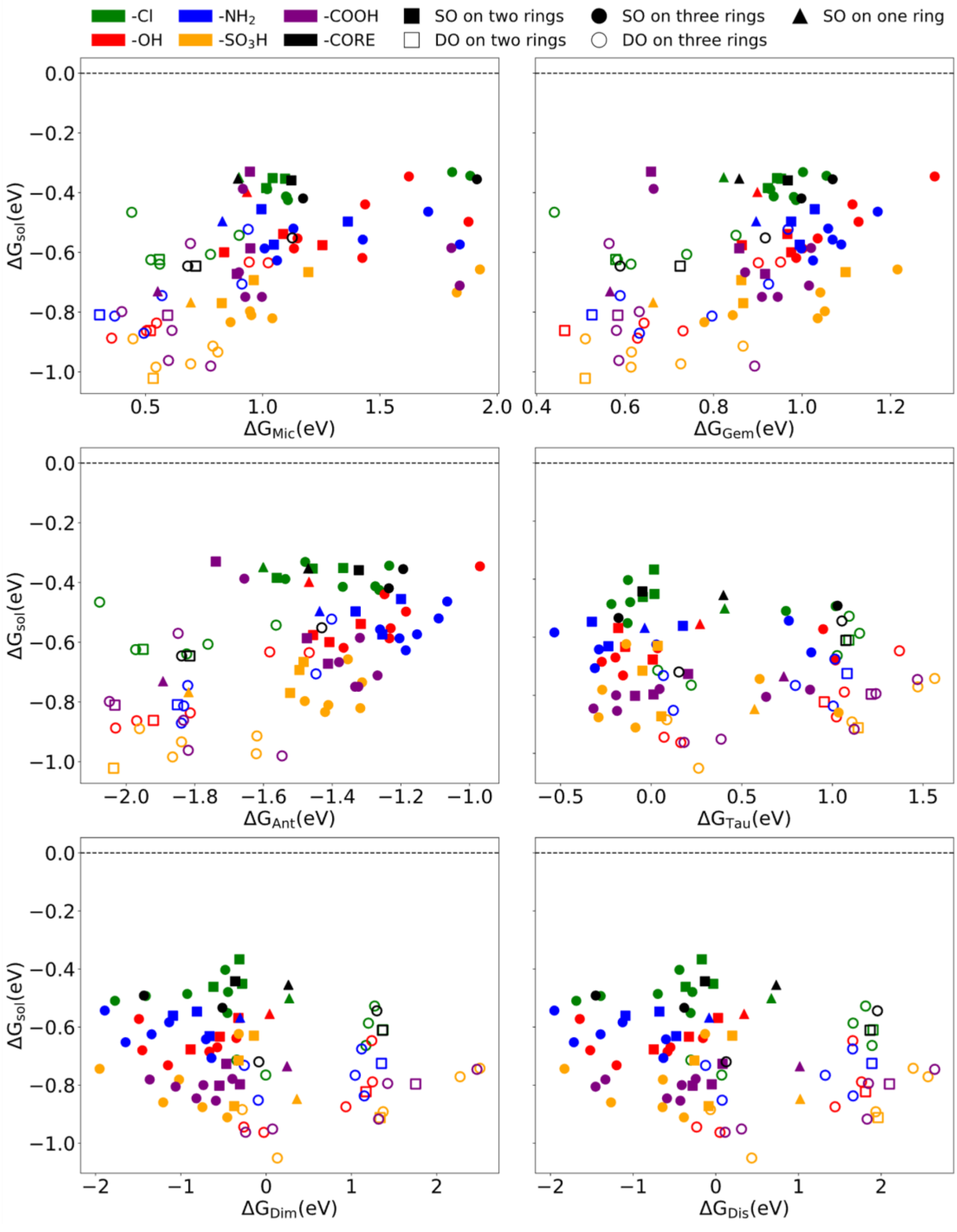

Figure S10. The relationship between the solvation reaction Gibbs free energy and the reaction Gibbs free energy of the six degradation mechanisms.

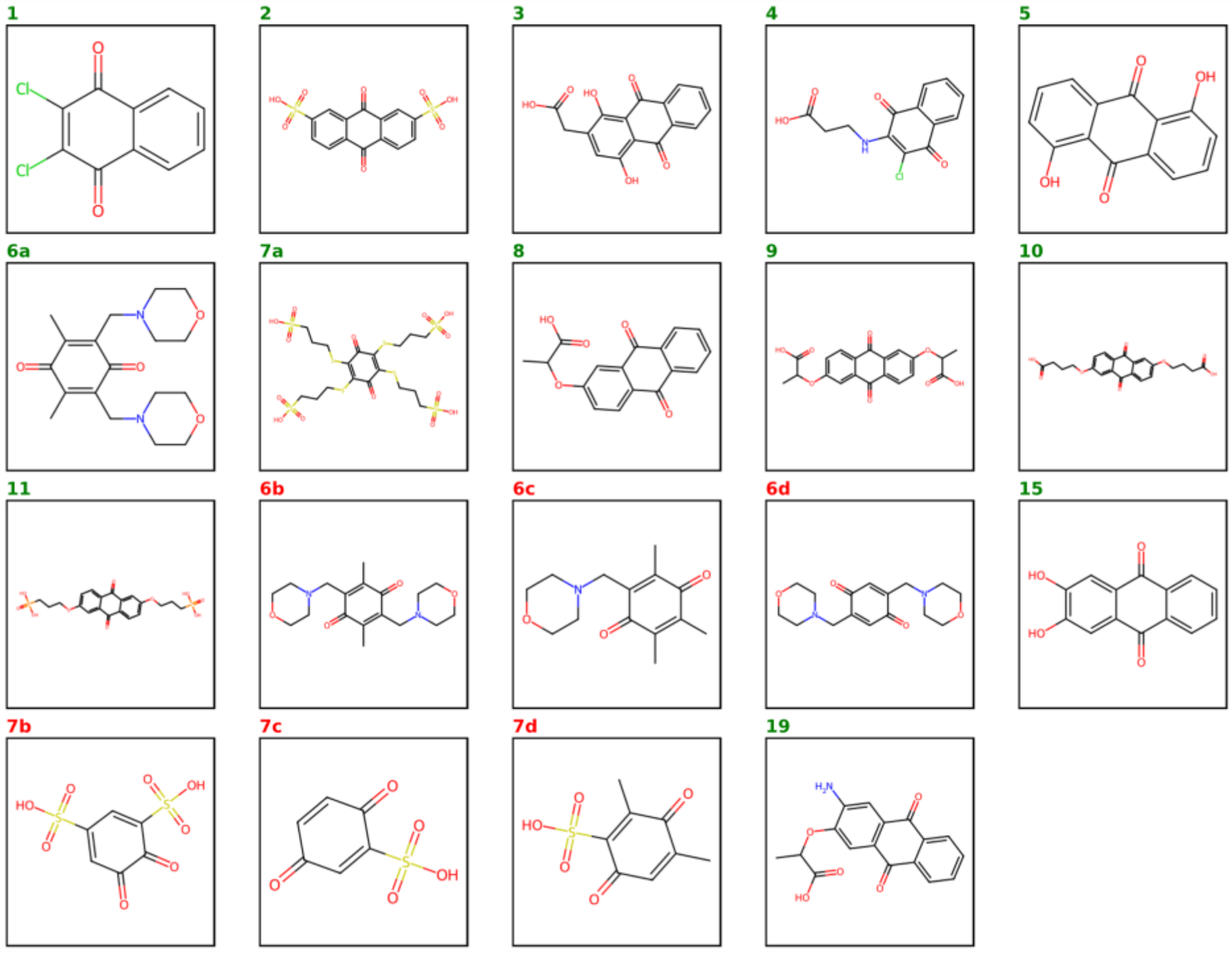

Figure S11. Structures of literature compounds used for validation. Label colour indicates relative cycling stability reported experimentally (green: more stable; red: less stable); see Table S3 for conditions and performance metrics.

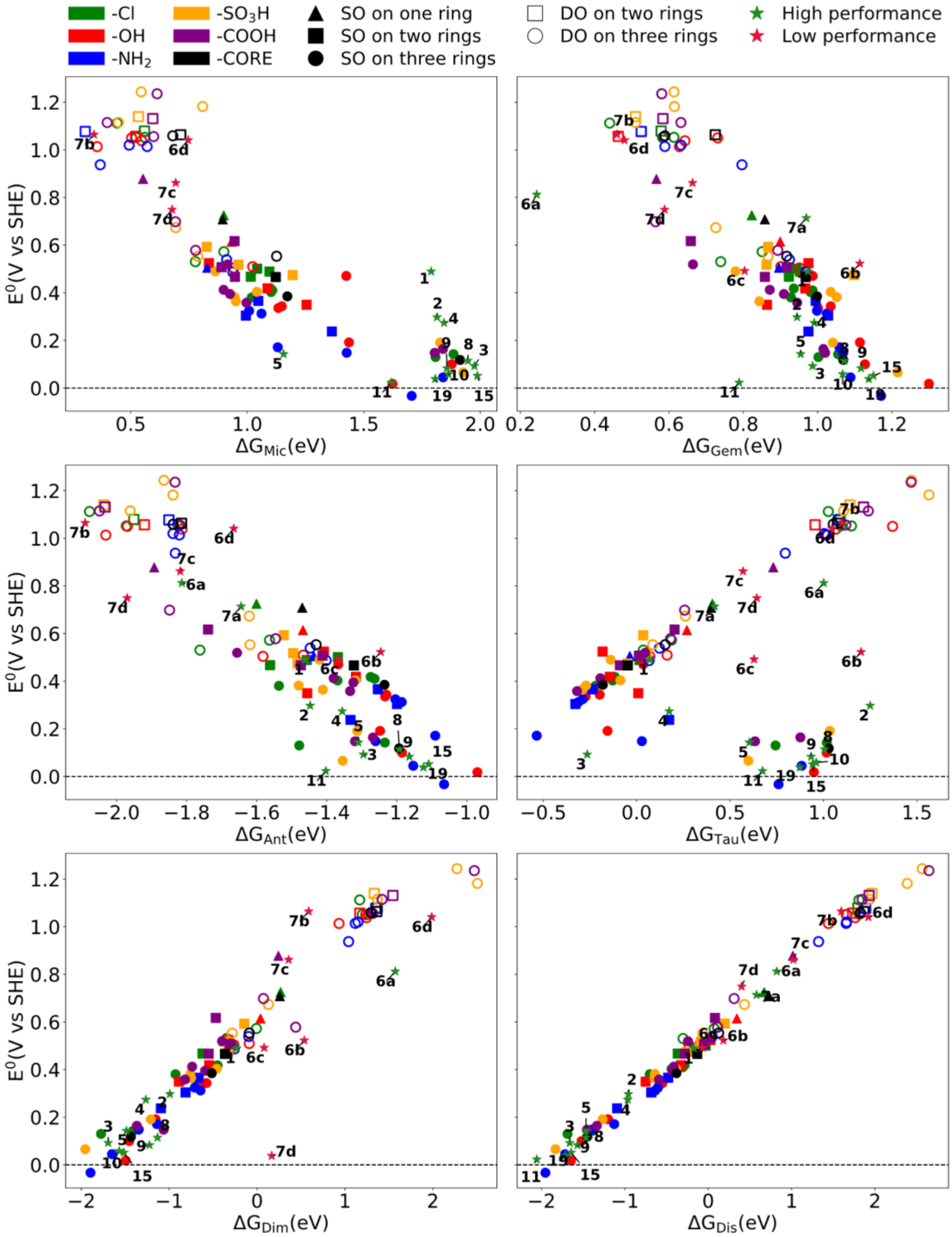

Figure S12. Experimental Validation of Theoretical Predictions: The relationship between the stability and the redox potential with the structures of the experimental points.

Table S2. A summary of 2D structures, SMILES representations, experimental redox potential(V versus SHE) of the compounds.

#	Molecule	SMILES	E°_{exp} (V vs. SHE)	Ref.
1	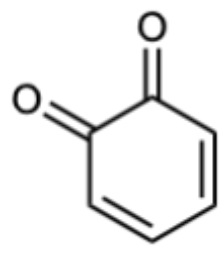	O=C1C(=O)C=CC=C1	0.831	[1]
2	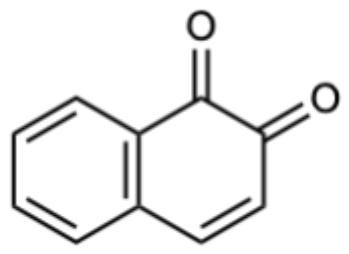	O=C1C(=O)C=Cc(c12)cccc2	0.547	[1]
3	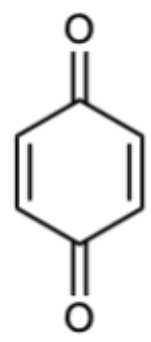	O=C1C=CC(=O)C=C1	0.699	[1]
4	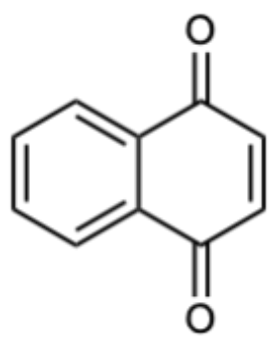	O=C1C=CC(=O)c(c12)cccc2	0.470	[1]
5	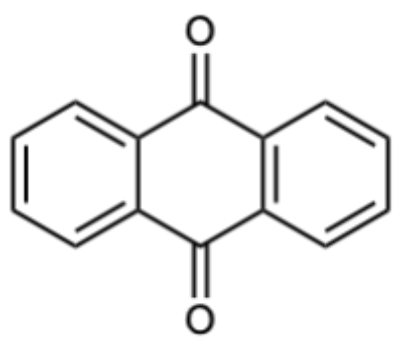	c1cccc(c12)C(=O)c3c(C2=O)cccc3	0.090	[1]
6	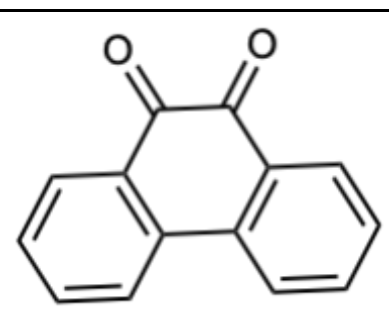	c1cccc(c12)c3c(C(=O)C2=O)cccc3	0.442	[1]
7	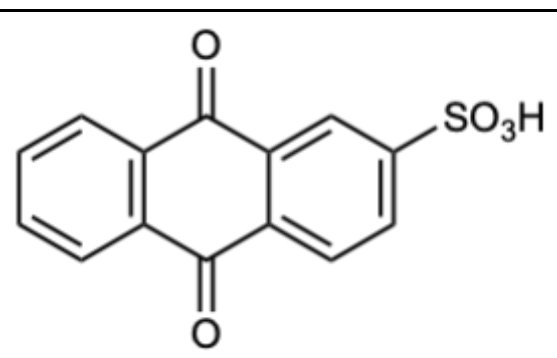	c1cccc(C2=O)c1C(=O)c(c23)cc(cc3)S(=O)(=O)O	0.187	[2]

8	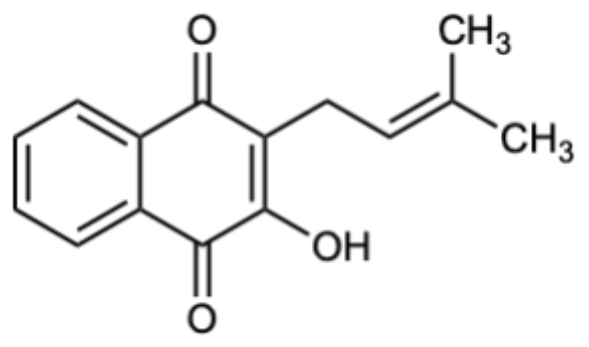	c1cccc(c12)C(=O)C(=C(C2=O)O)CC=C(C)C	0.333	[3]
9	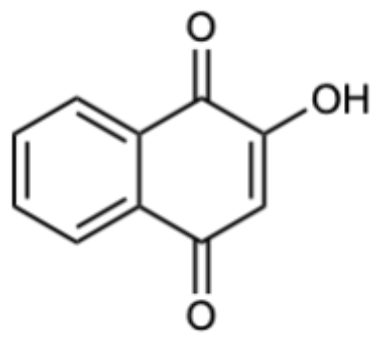	c1cccc(c12)C(=O)C(O)=CC2=O	0.308	[3]
10	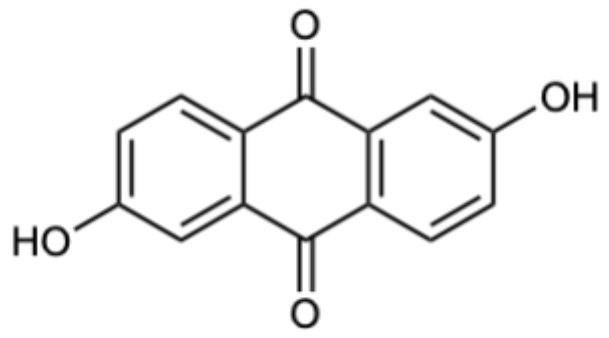	c1c(O)ccc(c12)C(=O)c3c(C2=O)ccc(c3)O	0.039	[3]
11	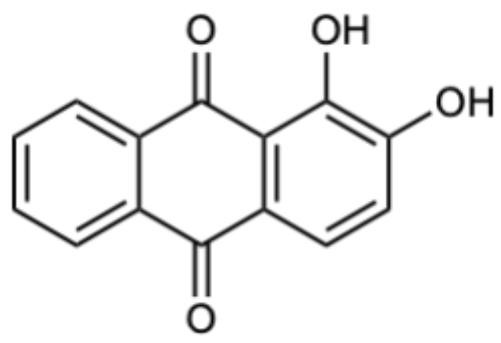	Oc1c(O)ccc(c12)C(=O)c3c(C2=O)cccc3	0.005	[3]
12	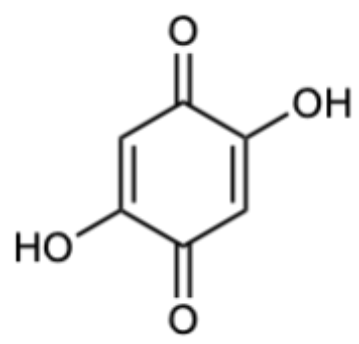	OC1=CC(=O)C(O)=CC1=O	0.382	[3]
13	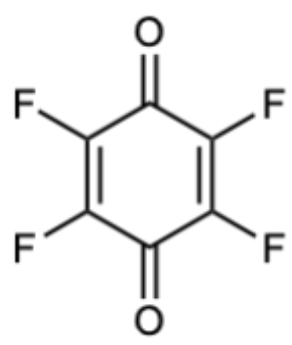	O=C1C(F)=C(F)C(=O)C(F)=C1F	0.707	[3]
14	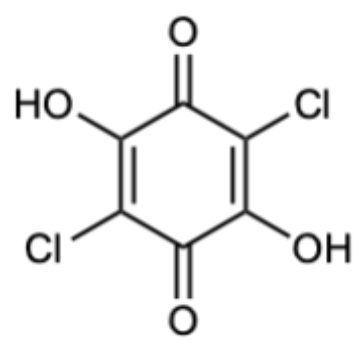	O=C1C(O)=C(Cl)C(=O)C(=C1Cl)O	0.394	[3]
15	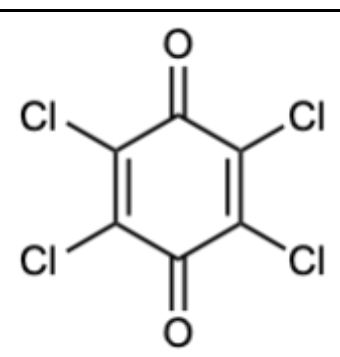	O=C1C(Cl)=C(Cl)C(=O)C(Cl)=C1Cl	0.700	[3]

16	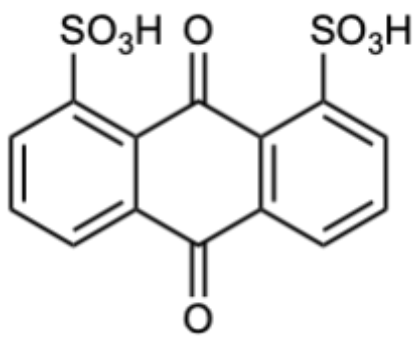	c1ccc(S(=O)(=O)O)c(c12)C(=O)c3c(C2=O)cccc3S(=O)(=O)O	0.206	[4]
17	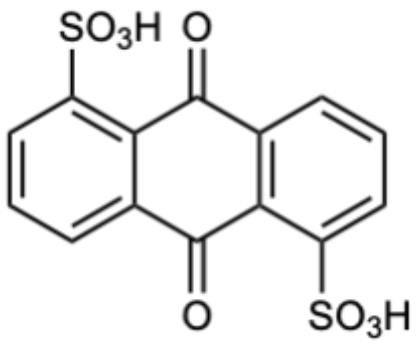	c1ccc(S(=O)(=O)O)c(c12)C(=O)c3c(C2=O)c(S(=O)(=O)O)ccc3	0.239	[4]
18	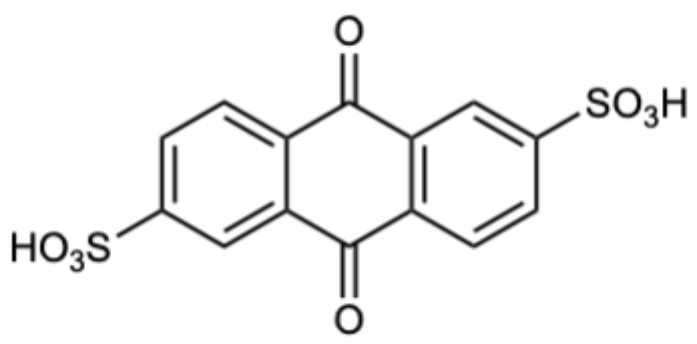	O=S(=O)(O)c(cc1)cc(c12)C(=O)c3c(C2=O)cc(S(=O)(=O)O)cc3	0.228	[4]
19	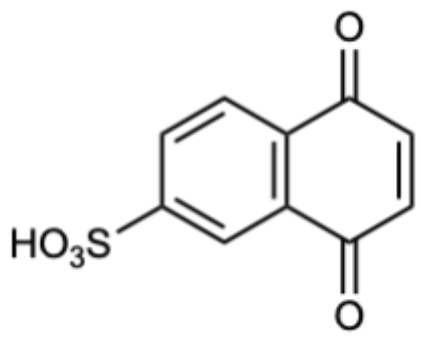	O=S(=O)(O)c(cc1)cc(c12)C(=O)C=CC2=O	0.534	[4]
20	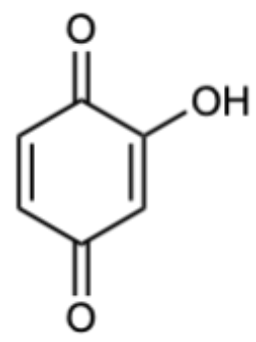	O=C1C=CC(=O)C(O)=C1	0.594	[4]
21	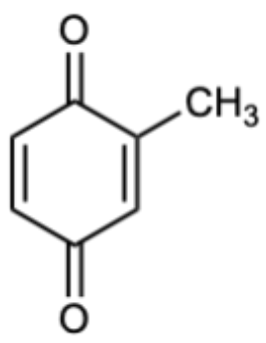	CC1=CC(=O)C=CC1=O	0.641	[4]
22	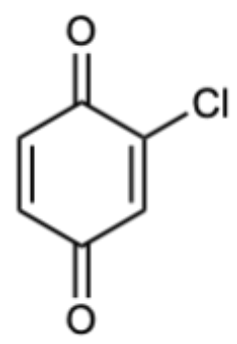	O=C1C=CC(=O)C(Cl)=C1	0.71	[4]

23	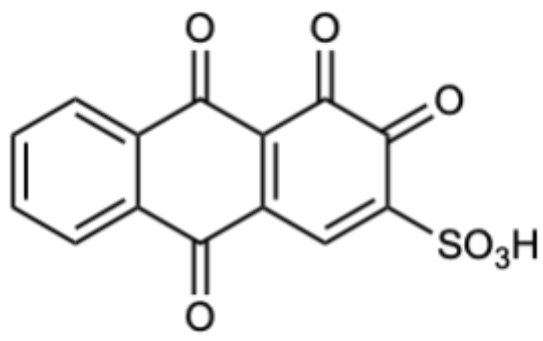	O=c1c(=O)c(S(=O)(=O)O)cc(c2=O)c1c(=O)c(c23)cccc3	1.21	[4]
24	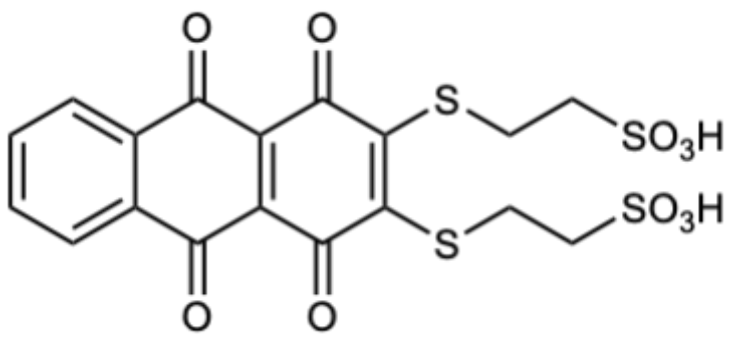	c1cccc(c12)c(=O)c3c(c2=O)c(=O)c(SCCS(=O)(=O)O)c(c3=O)SCCS(=O)(=O)O	1.08	[4]
25	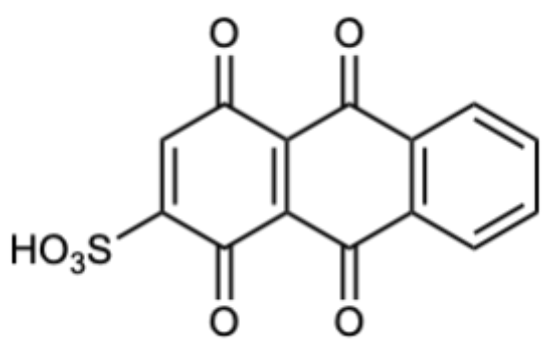	O=c1c(S(=O)(=O)O)cc(=O)c(c2=O)c1c(=O)c(c23)cccc3	1.05	[4]

Table S3. Summary of experimental redox couples and performance metrics of AORFBs from literature

ID	RFB positive/electrolyte/negative	pH	Current density	Cycle no.	Coulombic efficiency	Capacity fading rate	Capacity retention at cycle no.	Energy efficiency at cycle no.	Loss in discharge capacity	Discharge capacity	Charge efficiency
1[5]	2Cl-NQ/1 M H2SO4/PTCDA(perylene-3,4,9,10-tetracarboxylic dianhydride)	2.78	1 Ag^{-1}	1800	100%		98.60%				
2[6]	1,4-dihydroxyphenylsulfonate potassium (HQS)/Na2SO4/9,10-anthraquinone-2,7-disulfonic salt (2,7-AQDS)	7	60 $mAcm^{-2}$	120	96.00%			56%			
3[7]	K4Fe(CN)6/KOH/1,4-dihydroxy-2-carboxymethyl-9,10-anthraquinone (1,4-CDHAQ)	13	40 $mAcm^{-2}$	2500	97%	0.28% / day	55%	82%			

ID	RFB positive/electrolyte/negative	pH	Current density	Cycle no.	Coulombic efficiency	Capacity fading rate	Capacity retention at cycle no.	Energy efficiency at cycle no.	Loss in discharge capacity	Discharge capacity	Charge efficiency
4[8]	K3Fe(CN)6([Fe(CN)6]3−/4)/KOH / 1,5-dihydroxyanthraquinone(1,5-DHAQ-PAQS)	14	80 $mAcm^{-2}$	500	99%		94.30%	48.00%			
5[9]	ferrocyanide/NaOH/ sodium 3,3′,3″,3‴-((9,10-anthraquinone-2,6diyl)bis(azanetriyl))tetrakis(propane-1-sulfonate)(2,6-N-TSAQ)	14	40 $mAcm^{-2}$	900	99.9 %	0.025%/day					
6a[5]	2,6-dimethyl-3,5-bis(morpholinomethylene)benzene-1,4-diol (asym-O-5)/H2SO4/2,6-dimethyl-3,5-bis(morpholinomethylene)benzene-1,4-diol (asym-O-5)	1.54	20 $mAcm^{-2}$	300		1.8% /day					

ID	RFB positive/electrolyte/negative	pH	Current density	Cycle no.	Coulombic efficiency	Capacity fading rate	Capacity retention at cycle no.	Energy efficiency at cycle no.	Loss in discharge capacity	Discharge capacity	Charge efficiency
6b[5]	2,5-dimethyl-3,6-bis(morpholino methylene)benzene-1,4-diol (O-5)/H2SO4/2,5-dimethyl-3,6-bis(morpholinomethylene)benzene -1,4-diol (O-5)	1.54	50 $mAcm^{-2}$	300		0.87% /day					
6c[5]	2,3,5-trimethyl-6-(morpholino methylene)benzene-1,4-diol (O-1)/H2SO4/2,3,5-trimethyl-6-(morpholinomethylene)benzene-1,4-diol (O-1)	1.54	50 $mAcm^{-2}$	300		7.3% /day					
6d[5]	2,5-bis(morpholinomethylene)benzene -1,4-diol (O-3)/H2SO4/2,5-	1.54	50 $mAcm^{-2}$	300		52.7% /day					

ID	RFB positive/electrolyte/negative	pH	Current density	Cycle no.	Coulombic efficiency	Capacity fading rate	Capacity retention at cycle no.	Energy efficiency at cycle no.	Loss in discharge capacity	Discharge capacity	Charge efficiency
	bis(morpholinomethylene)benzene -1,4-diol (O-3)										
7a[10]	a tetrasubstituted quinone containing four sulfonated thioether substituents/H2SO4/anthraquinone -2,7-disulfonate (AQDS)	2.75	50 $mAcm^{-2}$	50				75%	2%	90%	100%
7b[10]	4,5-Dihydroxy-1,3-benzenedisulfonate/H2SO4/anthraquinone-2,7-disulfonate (AQDS)	2.75	50 $mAcm^{-2}$	50				50%	48%	50%	95%
7c[10]	para-Hydroxy benzenesulfonic acid/H2SO4/anthraquinone-2,7-disulfonate (AQDS)	2.75	50 $mAcm^{-2}$	50				40%	52%	30%	95%

ID	RFB positive/electrolyte/negative	pH	Current density	Cycle no.	Coulombic efficiency	Capacity fading rate	Capacity retention at cycle no.	Energy efficiency at cycle no.	Loss in discharge capacity	Discharge capacity	Charge efficiency
7d[10]	2,4-Dimethyl-3-sulfo-1,5-dihydroxybenzene/H2SO4/anthraquinone-2,7-disulfonate (AQDS)	2.75	50 $mAcm^{-2}$	50				70%	40%	20%	99.80%
8[11]	ferrocyanide/KCl/2-2PEAQ	7	40 $mAcm^{-2}$	2000	≈100%	0.09% /day					
	ferrocyanide/KOH/2-2PEAQ	14	40 $mAcm^{-2}$	800	≈100%	0.05% /day					
9[9]	ferro-/ferricyanide//2,2′-((9,10-dioxo-9,10-dihydroanthracene-2,6-diyl)bis(oxy))dipropionic acid (2,6-D2PEAQ)	7	100 $mAcm^{-2}$	170	99.70%	0.02% /day					
10[12]	potassium ferrocyanide//4,4′-((9,10-anthraquinone-2,6-	12	100 $mAcm^{-2}$	880		0.01% /day					

ID	RFB positive/electrolyte/negative	pH	Current density	Cycle no.	Coulombic efficiency	Capacity fading rate	Capacity retention at cycle no.	Energy efficiency at cycle no.	Loss in discharge capacity	Discharge capacity	Charge efficiency
	diyl)dioxy)dibutyrate (2,6-DBEAQ)										
11[13]	ferri/ferrocyanide//2,6-DPPEAQ, (((9,10-dioxo-9,10-dihydroanthracene-2,6-diyl)bis(oxy))bis(propane-3,1-diyl))bis(phosphonic acid)	9	100 $mAcm^{-2}$	480	99.90%	0.014% /day		65%			
15[14]	potassium ferrocyanide/NaOH/2,3-dihydroxylated anthraquinone(2,3-DHAQ)	14	50 $mAcm^{-2}$	3000	100.00%	0.8% / day		75%			
19[15]	ferrocyanide/NaOH/3-NH2-2-2PEAQ	14	80		>98%	0.01% / day		>70%			

Reference:

[1] Wass JRTJ, Ahlberg E, Panas I, et al. Quantum Chemical Modeling of the Reduction of Quinones. The Journal of Physical Chemistry A. 2006;110(5).

[2] Gerhardt MR, Tong L, Gómez-Bombarelli R, et al. Anthraquinone Derivatives in Aqueous Flow Batteries. Advanced Energy Materials. 2016;7(8).

[3] Wedege K, Drazevic E, Konya D, et al. Organic Redox Species in Aqueous Flow Batteries: Redox Potentials, Chemical Stability and Solubility. Sci Rep. 2016;6:39101.

[4] Tabor DP, Gómez-Bombarelli R, Tong L, et al. Mapping the frontiers of quinone stability in aqueous media: implications for organic aqueous redox flow batteries. Journal of Materials Chemistry A. 2019;7(20):12833-12841.

[5] Lu H, Yu J, Zhang J, et al. Designing p-π Conjugated Naphthoquinone-Derivatives as High-Performance Cathodes for Aqueous Proton Batteries. Advanced Functional Materials. 2025;35(24).

[6] Yang G, Zhu Y, Hao Z, et al. An Aqueous All-Quinone-Based Redox Flow Battery Employing Neutral Electrolyte. Advanced Energy Materials. 2024;14(26).

[7] Ozouf I, Fontmorin JM, Lebeuf R, et al. Long-cycling of a water-soluble quinizarin derivative in redox flow batteries: Role of the cut-off voltage on the stability. Electrochimica Acta. 2024;475.

[8] Liu Y, Zhang P, Wu Z, et al. Biomimetic Naphthoquinone Zwitterion Derivative with Water-Solubilizing Amino Acid Side Chain for High-Stability Aqueous Redox Flow Batteries. ACS Energy Letters. 2024;9(2):586-593.

[9] Wu M, Bahari M, Jing Y, et al. Highly Stable, Low Redox Potential Quinone for Aqueous Flow Batteries**. Batteries & Supercaps. 2022;5(6).

[10] Kwabi DG, Lin K, Ji Y, et al. Alkaline Quinone Flow Battery with Long Lifetime at pH 12. Joule. 2018;2(9):1894-1906.

[11] Guiheneuf S, Godet-Bar T, Fontmorin JM, et al. A new hydroxyanthraquinone derivative with a low and reversible capacity fading process as negolyte in alkaline aqueous redox flow batteries. Journal of Power Sources. 2022;539.

[12] Ding Y, Li Y, Yu G. Exploring Bio-inspired Quinone-Based Organic Redox Flow Batteries: A Combined Experimental and Computational Study. Chem. 2016;1(5):790-801.

[13] Wu M, Jing Y, Wong AA, et al. Extremely Stable Anthraquinone Negolytes Synthesized from Common Precursors. Chem. 2020;6(6):1432-1442.

[14] Yang X, Garcia SN, Janoschka T, et al. Novel, Stable Catholyte for Aqueous Organic Redox Flow Batteries: Symmetric Cell Study of Hydroquinones with High Accessible Capacity. Molecules. 2021;26(13).

[15] Alfaraidi AM, Xi D, Ni N, et al. An Extremely Stable and Soluble NH2-Substituted Anthraquinone Electrolyte for Aqueous Redox Flow Batteries. ACS Applied Energy Materials. 2023;6(24):12259-12266.
